# De novo synthesis of cardiolipin controls respiratory chain biogenesis in neonatal mouse hearts

**DOI:** 10.1038/s44319-026-00864-8

**Published:** 2026-07-07

**Authors:** Mindong Ren, Yang Xu, Colin K L Phoon, Hediye Erdjument-Bromage, Thomas A Neubert, Michael Schlame

**Affiliations:** 1 Department of Anesthesiology, New York University Grossman School of Medicine, New York, NY USA; 2 Department of Pediatrics, New York University Grossman School of Medicine, New York, NY USA; 3 Department of Neuroscience and Physiology, New York University Grossman School of Medicine, New York, NY USA

**Keywords:** Cardiovascular System, Metabolism, Organelles

## Abstract

Postnatal maturation of the mammalian heart requires a vast increase in respiratory enzymes. The mitochondria-specific lipid cardiolipin (CL) is essential for respiratory chain integrity but has no defined function in heart maturation. Here, we determined how the two steps of CL biogenesis, de novo synthesis and acyl chain remodeling, affect the maturation of cardiac mitochondria in mice. Cardiomyocyte-restricted deletion of the CL synthase *Crls1* in late gestation does not affect CL levels at birth but blocks the increase in the tissue concentration of CL observed during normal postnatal maturation. Deletion of *Crls1* prevents the postnatal rise in cristae density and in the intramitochondrial concentration of respiratory proteins. This inhibits cardiac development, precipitates heart failure, and causes death by the age of 2 weeks. In contrast, ablation of CL remodeling by cardiomyocyte-restricted deletion of *Tafazzin* does not disrupt mitochondrial maturation or cardiac development, although it has a similar effect on the CL concentration and profoundly alters the CL species composition. Our data show that CL synthesis, but not CL remodeling, controls expression of the respiratory chain by a mechanism independent of the CL concentration.

## Synopsis

Cardiolipin synthesis, but not cardiolipin remodeling, controls crista biogenesis and expression of the respiratory chain.

- Cardiomyocyte-restricted deletion of *Crls1* prevents the postnatal rise in cristae density and intra-mitochondrial concentration of respiratory proteins.
- Cardiomyocyte-restricted deletion of *Tafazzin* does not disrupt mitochondrial maturation although it has a similar effect on the CL concentration.
- Deletion of *Crls1*, but not *Tafazzin*, inhibits postnatal development of the heart causing premature death.

## Abstract (web summary)

Cardiolipin synthesis, but not cardiolipin remodeling, controls crista biogenesis and expression of the respiratory chain.

## Graphical abstract

**Figure:**
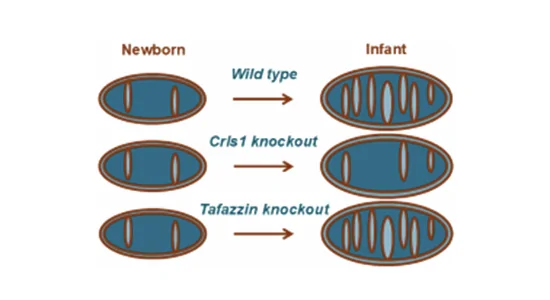

**Figure:**
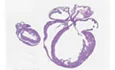

## Introduction

Mammalian heart development can be divided into three phases. The first phase, called specification, differentiates mesodermal progenitors into cardiac-specific lineages. The second phase, called morphogenesis, creates and connects the structural components of the heart. The third and final phase, called maturation, conditions the heart for lifelong contractions to sustain uninterrupted circulation. Maturation begins around birth and continues into the neonatal period. It alters cardiomyocytes through a tightly scripted program affecting all cellular compartments. Mitochondria, in particular, undergo profound changes in morphology and function, but the mechanisms that control mitochondrial maturation are incompletely understood (Dimasi et al, 2023; Guo and Pu, 2020; Sakamoto and Kelly, 2024).

Cardiolipin (CL) is the signature lipid of mitochondria. It is localized in the inner membrane where it creates the proper environment for the assembly and the function of the protein complexes of oxidative phosphorylation (Acoba et al, 2020; Mejia and Hatch, 2016; Ren et al, 2014). As a result, CL increases respiratory activity and the efficiency of energy transduction (Claypool et al, 2008; Xu et al, 2021), which makes it indispensable for tissues with high energy expenditure, like the heart. Indeed, CL was discovered in and named after lipid extracts of cardiac tissue (Pangborn, 1942). CL deficiency is associated with cardiomyopathy in patients with Barth syndrome (Schlame et al, 2003; Vreken et al, 2000), in patients with *Crls1* mutations (Lee et al, 2022), and in diabetic mice (Han et al, 2005; 2007). However, despite evidence that CL is essential for cardiac function, its specific role in cardiac maturation has not been studied.

CL is synthesized from phosphatidic acid at the inner leaflet of the inner membrane of mitochondria (Schlame and Haldar, 1993). The pathway requires four enzymes, including phosphatidate cytidylyltransferase (TAMM41), CDP-diacylglycerol:glycerol-3-phosphate 3-phosphatidyltransferase (PGS1), phosphatidylglycerophosphatase (PTPMT1), and cardiolipin synthase (CRLS1) (Ren et al, 2014). De novo synthesis is followed by fatty acid remodeling, which exchanges the acyl chains for unsaturated fatty acids. Remodeling is initiated by the lipase ABHD18 that removes fatty acids from CL (Ren et al, 2025; Masud et al, 2025). New and mostly unsaturated fatty acids are then transferred from other phospholipids to monolyso-cardiolipin (MLCL) by the acyltransferase TAFAZZIN (Xu et al, 2006). This step regenerates CL and changes its composition of molecular species.

To investigate the role of CL in pre- and postnatal development, several mouse models have been created (Olivar-Villanueva et al, 2023). Constitutive knockout (KO) of the remodeling enzyme *Tafazzin* increases perinatal lethality and causes dilated cardiomyopathy in adults, recapitulating the phenotype of human Barth syndrome (Wang et al, 2020). In contrast, constitutive KO of enzymes of the de novo pathway results in early embryonic death (Kasahara et al, 2020; Zhang et al, 2011). Viable mouse strains have been created though by tissue-specific deletions of *Crls1* and *Ptpmt1* in brown adipocytes (Sustarsic et al, 2018), neurons (Kasahara et al, 2020), and T-cells (Corrado et al, 2020). Those models have confirmed previously known effects of CL deficiency and uncovered novel tissue-specific functions in thermogenesis control and T-cell differentiation, but they have not been able to address the specific function of CL in the heart. To close this gap, a cardiomyocyte-restricted KO of *Ptpmt1* has been established (Chen et al, 2021). This induced CL deficiency in embryonic mouse hearts but again resulted in prenatal lethality. Cardiomyocyte-restricted KO of *Tafazzin* has also been accomplished and shown to mimic the cardiac phenotype of Barth syndrome (Wang et al, 2020; Zhu et al, 2021).

Here, we have created a viable mouse model with cardiomyocyte-restricted deletion of *Crls1* and compared it side-by-side to a model with cardiomyocyte-restricted deletion of *Tafazzin* on the same genetic background. Our data show that CL synthesis, but not CL remodeling, asserts control over cardiac maturation by regulating the expression of respiratory proteins.

## Results and discussion

### *Crls1*KO but not *Tafazzin*KO causes dilated cardiomyopathy and death in newborn mice

**Figure 1:**
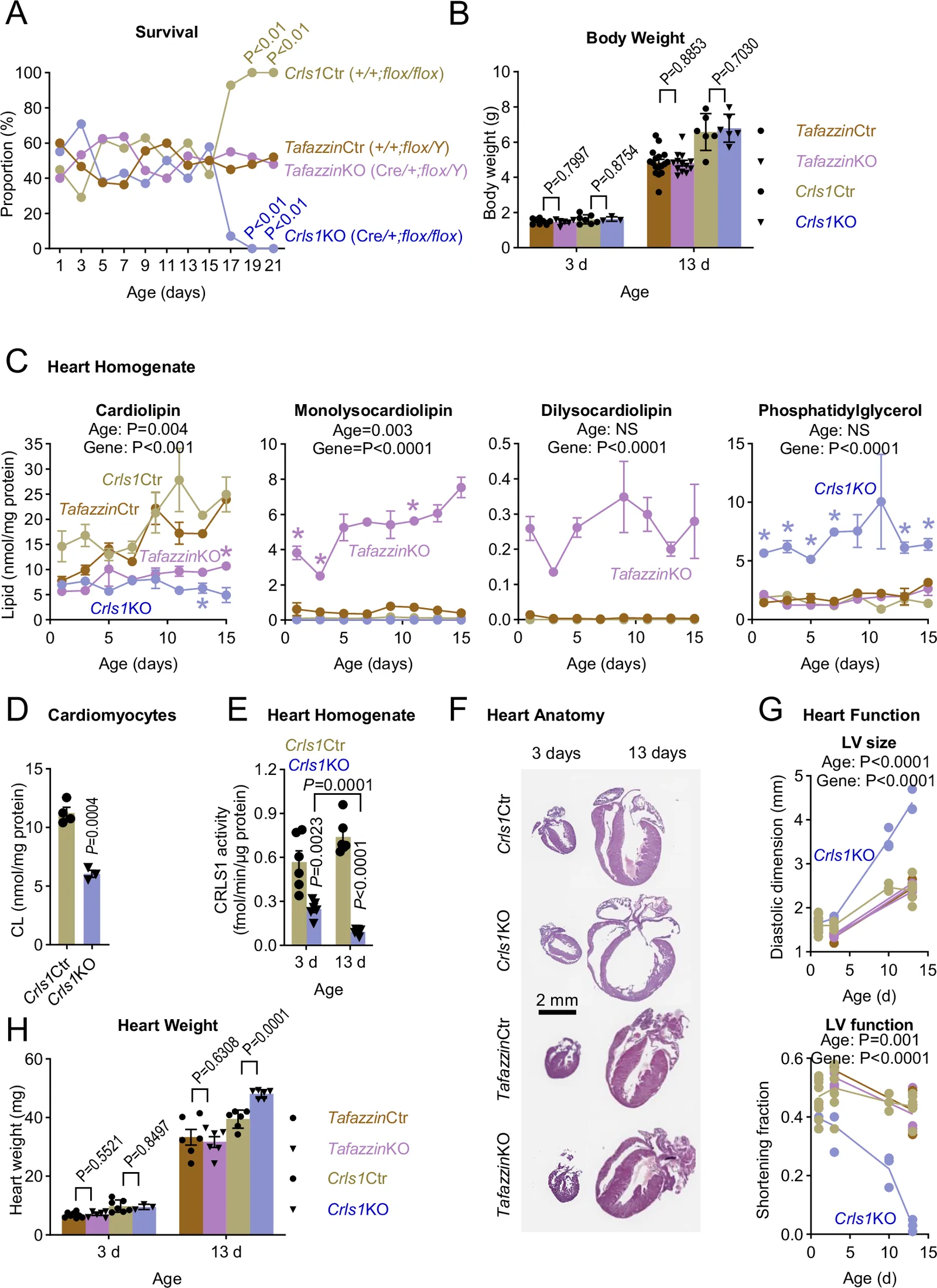
Phenotypes caused by cardiomyocyte-restricted deletions of *Tafazzin* and *Crls1.* (**A**) Male mouse pups were genotyped at different postnatal ages to determine the proportion of KOs and controls among *flox/flox* or *flox/Y* mice (19 ≤ *N* ≤ 30 for each time point). (**B**) Body weight of mouse pups was determined at postnatal ages of 3 and 13 days (3 ≤ *N* ≤ 17). (**C**) The indicated phospholipids were quantified in heart homogenates at different postnatal ages (*N* = 3 per time point). (**D**) CL was quantified in purified cardiomyocytes from 3-day-old mice (*N* = 4). (**E**) CRLS1 activity was measured in heart homogenate at postnatal ages of 3 and 13 days (*N* = 6). (**F**) Sections of mouse hearts were stained with hematoxylin and eosin. (**G**) The left ventricle (LV) of mouse hearts was analyzed by echocardiography (3 ≤ *N* ≤ 12). (**H**) Heart weight of mouse pups was determined at postnatal ages of 3 and 13 days (3 ≤ *N* ≤ 8). Graphs show means with standard deviations. Data were analyzed by two-tailed, binomial test (**A**), unpaired, two-tailed *t* test (**B**, **D**, **E**, **H**), or mixed-effects analysis using the restricted maximum likelihood method (**C**, **G**). Asterisks indicate a significant difference between KO and control (*P* < 0.05) after adjustment by the Bonferroni method. NS not significant. Source data are available online for this figure.

To determine the effect of CL on cardiac development, we inactivated either *Tafazzin* or *Crls1* in cardiomyocytes using *Cre-Lox* recombination with the *Myh6-Cre* driver. *Myh6-Cre/+*; *Tafazzin*^*flox/Y*^ (*Tafazzin*KO) and *Myh6-Cre/+*; *Crls1*^*flox/flox*^ (*Crls1*KO) mice were compared to their littermate controls (*Tafazzin*Ctr: +/+;*Tafazzin*^*flox/Y*^ and *Crls1*Ctr: +/+;*Crls1*^*flox/flox*^, respectively) and to each other. All genotypes were born at the expected Mendelian ratios, demonstrating that the cardiomyocyte-specific knockouts, which were induced late in cardiac morphogenesis via the *Myh6* promoter (Lyons et al, 1990), did not cause any prenatal lethality. However, *Crls1*KO mice died at the age of 16 days, whereas *Tafazzin*KO mice progressed normally through the neonatal period (Fig. 1A). Neither *Crls1*KO nor *Tafazzin*KO affected body weight up to an age of 13 days (Fig. 1B). Because *Tafazzin* is an X-linked gene, only male mice are affected. Sex did not affect survival in *Crls1*KO mice (*P* = 0.7031 by analysis of variance). Therefore, we limited the entire study to male mice.

At birth, the tissue concentrations of CL in the two KOs were not significantly different from those of their controls. During postnatal maturation, the CL concentration rapidly increased, but not in either the *Crls1*KO or the *Tafazzin*KO. In controls, CL more than doubled by the age of 15 days, whereas it remained almost unchanged in the mutants. *Tafazzin*KO also increased the concentrations of partially degraded CLs, such as MLCL and dilyso-cardiolipin (DLCL), whereas *Crls1*KO increased the concentration of the CL precursor phosphatidylglycerol (PG). These findings reflect the different causes of CL deficiency in the two mutants, namely excessive CL degradation in *Tafazzin*KO and insufficient CL synthesis in *Crls1*KO (Fig. 1C).

We were surprised to find CL in the hearts of *Crls1*KO pups. To verify that CL was contributed by cardiomyocytes, rather than fibroblasts or endothelial cells that are present in cardiac tissue, we purified cardiomyocytes from 3-day-old mouse hearts and determined their lipid composition. *Crls1*KO cardiomyocytes contained about half the normal CL concentration (Fig. 1D), showing that cardiomyocytes were the source of CL. The presence of CL in *Crls1*KO cardiomyocytes raised the question of how much CRLS1 was actually deleted.

**Figure EV1:**
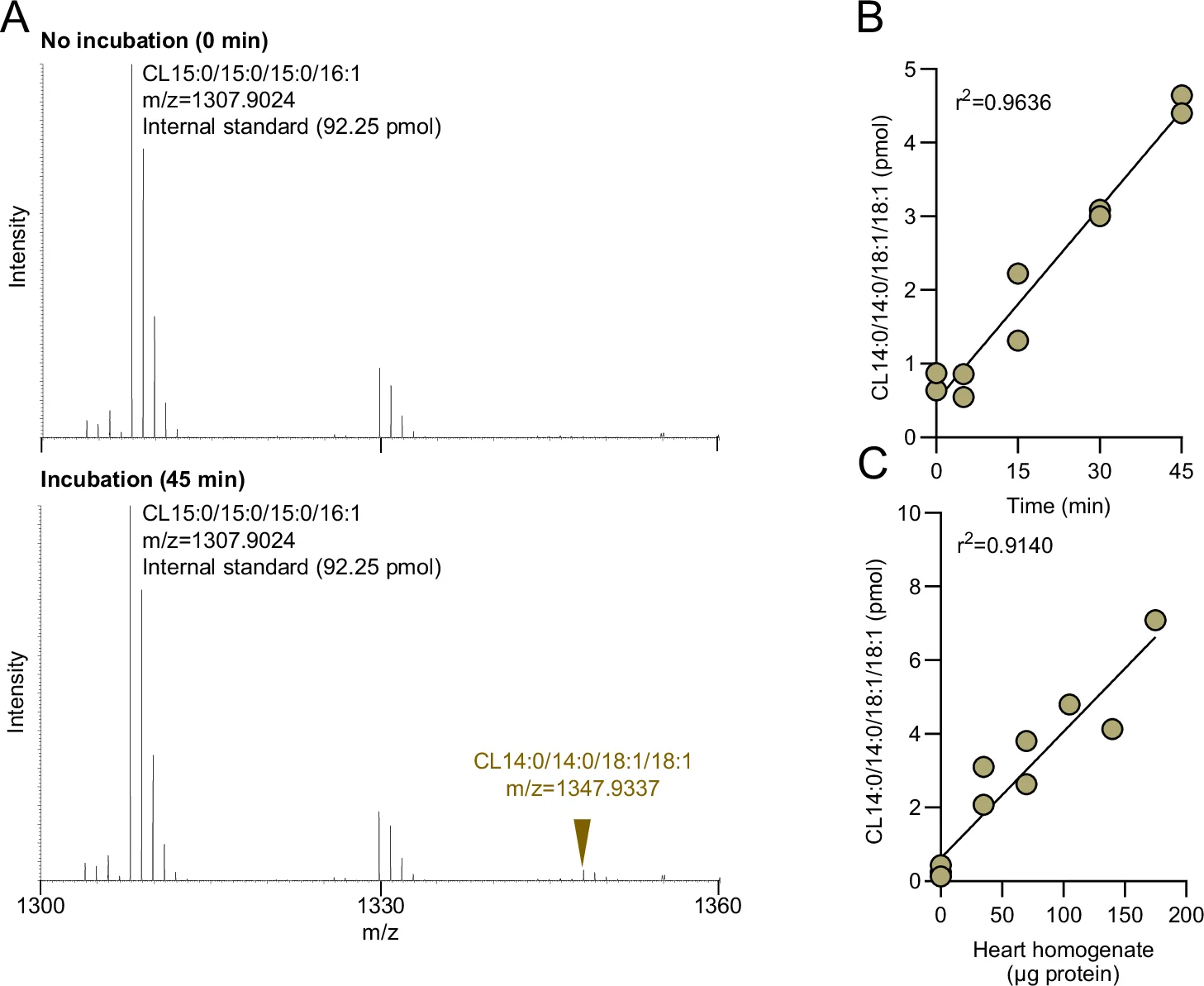
Assay of cardiolipin synthase (CRLS1) activity in heart homogenate. Mouse heart homogenate was incubated at 37 °C with dimyristoyl-phosphatidylglycerol (PG14:0/14:0) and CDP-dioleoylglycerol (CDPDG18:1/18:1) at pH 8 in the presence of Co^2+^. The formation of CL14:0/14:0/18:1/18:1 was measured by LC–MS with internal standards. (**A**) CL14:0/14:0/18:1/18:1 is not an endogenous species of mouse heart but becomes detectable upon incubation with CRLS1 substrates. The panel shows sub-spectra (*m/z* 1300–1360 Th, retention time 12–15 min) that contain the reaction product CL14:0/14:0/18:1/18:1 and the internal standard CL 15:0/15:0/15:0/16:1. The blank signal corresponded to 0.549 ± 0.266 pmol CL14:0/14:0/18:1/18:1 (*N* = 4). (**B**) CL14:0/14:0/18:1/18:1 increases at a constant rate during incubation. Assay mixtures contained 10 mM Tris (pH 8), 5 mM CoCl_2_, 0.1 mM PG14:0/14:0, 0.1 mM CDPDG18:1/18:1, and 70 µg heart protein in a total volume of 100 µL. Reactions were stopped after different time intervals. Lipids were extracted, and CL was quantified by LC–MS. Linear regression analysis was performed. (**C**) Formation of CL14:0/14:0/18:1/18:1 is linearly dependent on the amount of heart homogenate. Assay mixtures contained 10 mM Tris (pH 8), 5 mM CoCl_2_, 0.1 mM PG14:0/14:0, 0.1 mM CDPDG18:1/18:1, and different amounts of heart homogenate in a total volume of 100 µL. Reactions were stopped after 30 min. Lipids were extracted, and CL was quantified by LC–MS. Linear regression analysis was performed.

To determine KO efficiency, we developed an assay for the enzymatic activity of CRLS1 (Fig. EV1). *Crls1*KO reduced the enzymatic activity in newborn mouse hearts only by 50–60% but caused >85% reduction by the age of 13 days (Fig. 1E). Thus, *Crls1*KO hearts retained some CRLS1 activity at birth, which was sufficient to sustain low concentrations of CL in the growing heart.

Early lethality of *Crls1*KO mice could be attributed to heart failure. Although no cardiac defects were detectable at birth, severe dilated cardiomyopathy developed in the neonatal period as demonstrated by thinned ventricular walls (Fig. 1F), increased diastolic dimensions, and reduced shortening fractions (Fig. 1G). By the age of 13 days, the weight of *Crls1*KO hearts was 22% higher than normal (Fig. 1H). In contrast, cardiac morphology, function, and weight of *Tafazzin*KO mice were indistinguishable from those of controls.

In summary, cardiomyocyte-restricted KO of either *Tafazzin* or *Crls1* did not affect the CL concentration at birth but prevented the rise of CL during postnatal cardiac maturation. This was caused by accelerated CL degradation in *Tafazzin*KO hearts and by insufficient CL synthesis in *Crls1*KO hearts. However, only *Crls1*KO induced a dilated cardiomyopathy that led to 100% lethality before weaning.

### Both *Tafazzin*KO and *Crls1*KO affect the postnatal maturation of the cardiac lipidome

To track the postnatal development of the cardiac lipidome, we collected mouse hearts at 2-day intervals and analyzed their lipid extracts by liquid chromatography/tandem mass spectrometry (LC–MS/MS). In each strain, we measured the relative abundance of about a thousand lipid species (779–1255) and quantified most of them (674–1025) with internal standards. Normal postnatal development of the heart was associated with substantial changes in the lipid composition. Phosphatidylcholine (PC), phosphatidylethanolamine (PE), and sphingomyelin (SM) species were dominant among lipids that changed their concentration with postnatal age as revealed by correlation analysis (Dataset EV1).

**Figure 2:**
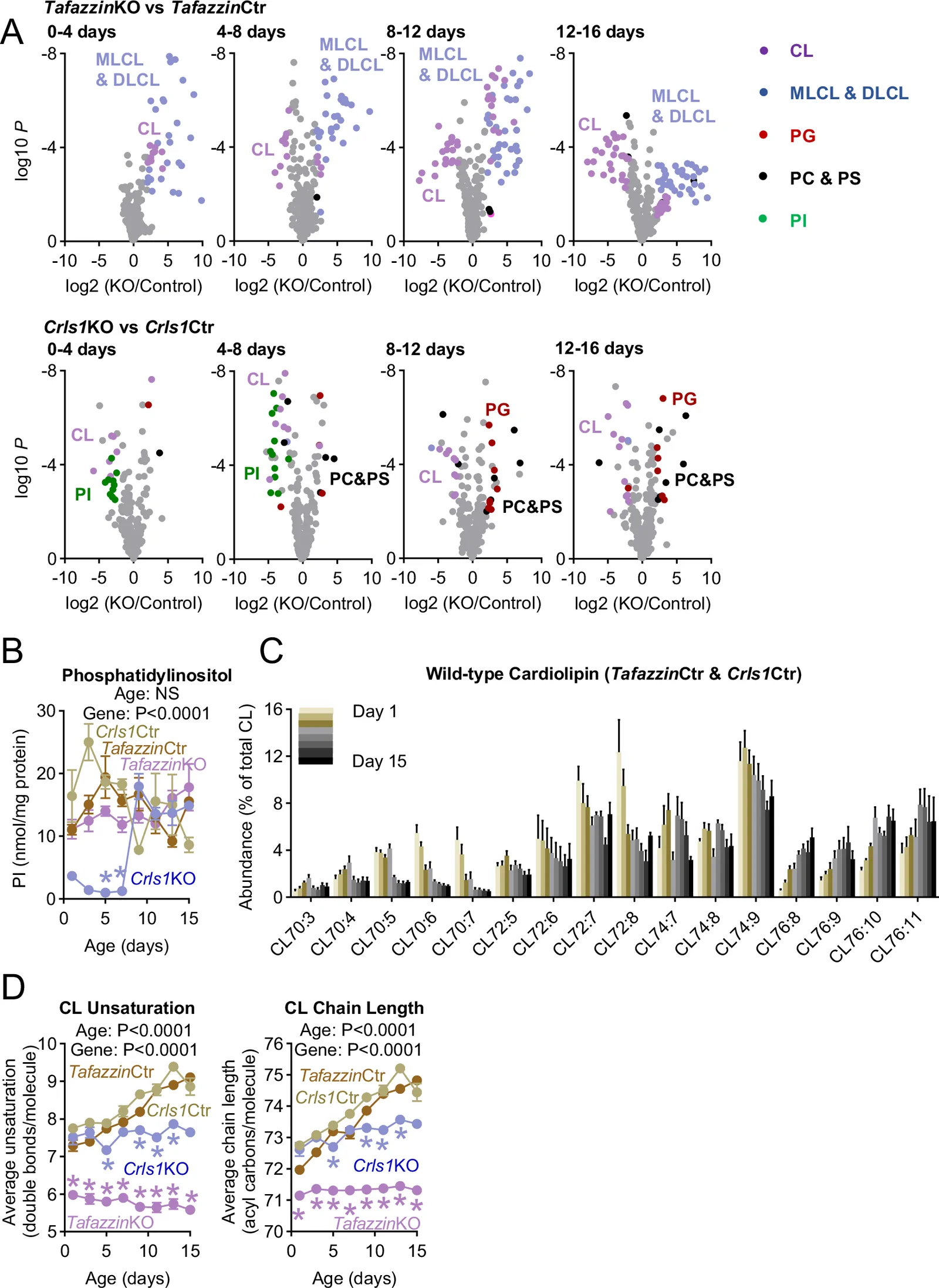
Effects of *Tafazzin*KO and *Crls1*KO on the postnatal development of the cardiac lipidome. (**A**) Lipids were analyzed in heart homogenates. Volcano plots were created from measurements in 6 biological replicas per genotype and age group (pooled data from two time points per group). *P* value thresholds were determined by the Benjamini–Hochberg procedure assuming a false discovery rate of 0.05. Lipid species with significant differences between genotypes (fourfold higher or lower, *P* < 0.01) are shown in color. (**B**–**D**) Total phosphatidylinositol and individual CL species were quantified in heart homogenates at different postnatal ages (*N* = 3 per age). (**C**) Shows abundant molecular groups of CL (> 2% of total CL). Data are means with standard deviations. Mixed-effects analysis was performed using the restricted maximum likelihood method (**B**, **D**) to determine *P* values for the effects of age and genotype. Asterisks indicate a significant difference between KO and the corresponding control (*P* < 0.05) after adjustment by the Bonferroni method. NS not significant.

To identify the effects of the two genes on lipidome development, we created a series of age-specific volcano plots. Deletion of *Tafazzin* caused changes in CL, MLCL, and DLCL. Those changes became larger with increasing postnatal age. Deletion of *Crls1* had a much broader impact on the lipidome, affecting phosphatidylinositol (PI), PC, and phosphatidylserine (PS), in addition to CL and PG (Fig. 2A). With increasing age, more PC and PS species changed their concentration. The total PI concentration was abnormally low at birth but increased back to normal by postnatal day 9 (Fig. 2B).

**Figure EV2:**
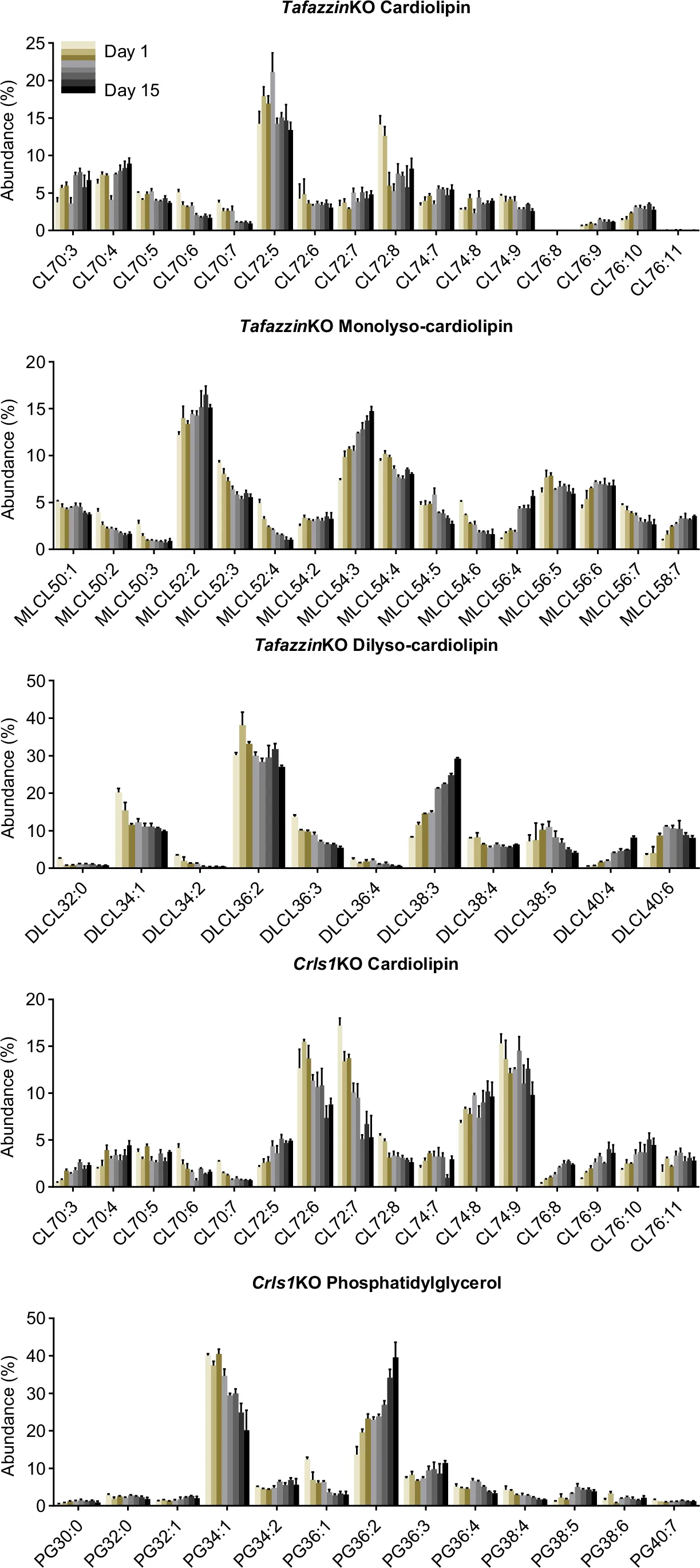
Age-dependent species patterns of CL, MLCL, DLCL, and PG in *Tafazzin*KO and *Crls1*KO. Lipids were analyzed in heart homogenates at different postnatal ages. Bar graphs show means with standard deviations (*N* = 3) of abundant molecular groups (> 2% of phospholipid class).

In controls, postnatal maturation led to drastic changes in the molecular species composition of CL. For instance, tetralinoleoyl-CL (CL72:8) was very abundant at birth but decreased during maturation. However, most long-chain, polyunsaturated species increased with postnatal age while the relative abundance of shorter and more saturated species decreased (Fig. 2C). This led to a gradual rise in unsaturation and chain length during normal maturation. Deletion of *Tafazzin* caused a reduction of unsaturation and chain length at birth and prevented their age-dependent increase. Deletion of *Crls1* did not affect these parameters at birth but inhibited the postnatal increase in unsaturation and chain length (Figs. 2D and  EV2). Metabolites that accumulated in deletion strains, such as MLCL, DLCL, and PG, also underwent small changes in their molecular species patterns during postnatal maturation (Fig. EV2).

In summary, the characteristic species composition of heart CL emerged gradually during the neonatal period. Deletion of *Tafazzin* prevented CL maturation, as expected, while deletion of *Crls1* slowed down the maturation of the remaining CL. In addition, *Crls1* deletion caused large changes in the lipidome affecting PI, PC, and PS.

### *Crls1*KO but not *Tafazzin*KO inhibits the postnatal accumulation of cristae and respiratory enzymes

To track the postnatal maturation of the cardiac proteome, we analyzed protein compositions of mouse hearts in 2-day intervals. Using label-free relative quantitative proteomics, we identified over 5000 proteins in the hearts of newborn mice, which belonged to the nucleus (30%), mitochondria (15%), plasma membrane (15%), cytosol (11%), endoplasmic reticulum (8%), myofibrils (8%), and others. In control mice, the abundance of many mitochondrial proteins showed a strong positive correlation with age (Dataset EV2).

**Figure 3:**
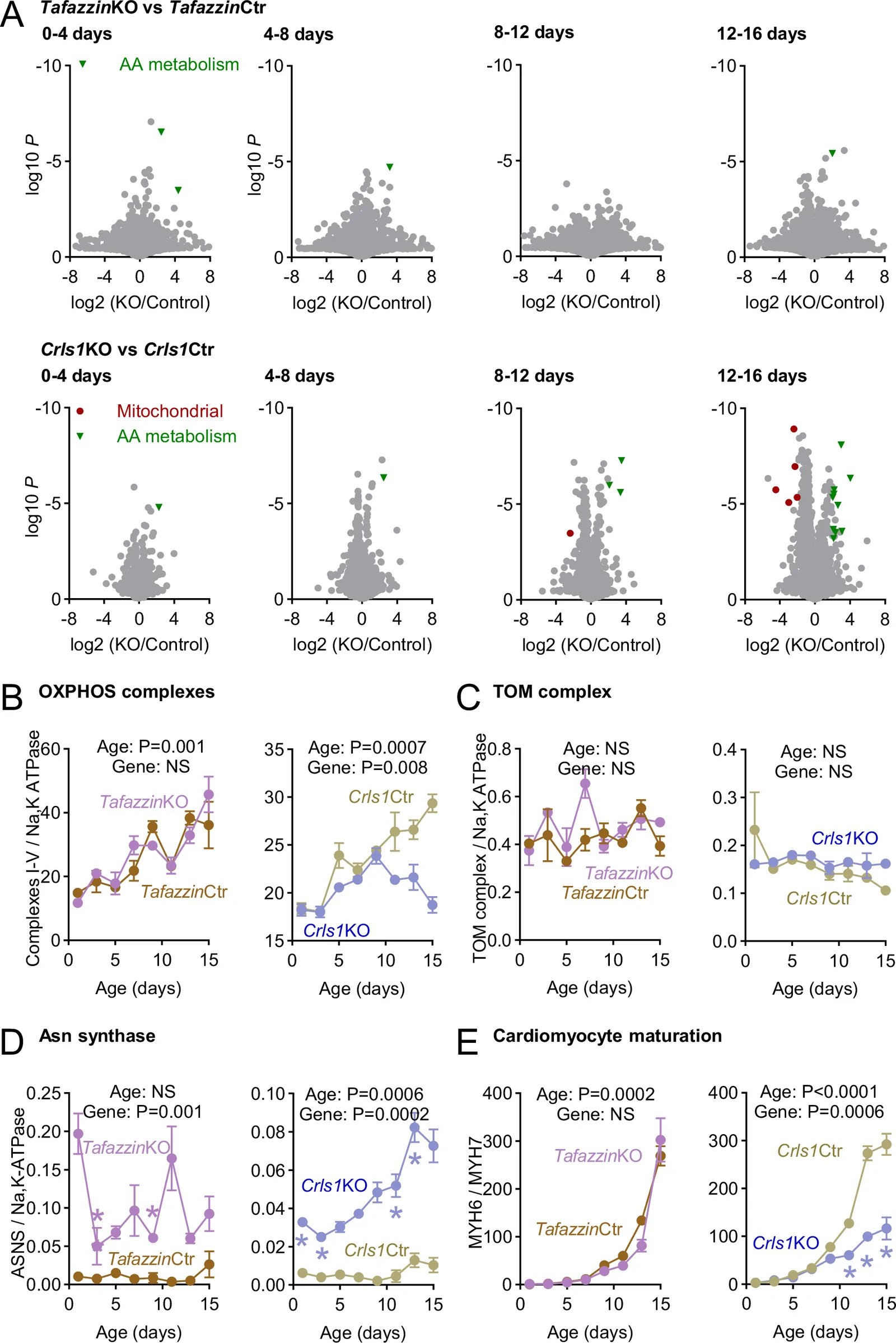
Effects of *Tafazzin*KO and *Crls1*KO on the postnatal development of the cardiac proteome. (**A**) Proteins were analyzed in heart homogenates. Volcano plots were created from measurements in six biological replicas per genotype and age group (pooled data from two time points per group). *P* value thresholds were determined by the Benjamini–Hochberg procedure assuming a false discovery rate of 0.05. Enzymes of amino acid metabolism and mitochondrial proteins with significant differences between genotypes (fourfold higher or lower, *P* < 0.001) are shown in color. (**B**–**E**) Proteins were analyzed in heart homogenates. Data (means with standard deviations, *N* = 3 per age) are ratios of composite protein abundances. To calculate the abundance of a protein complex or a group of proteins, LFQ intensities of individual subunits (listed in Dataset EV2) were added. Mixed-effects analysis was performed using the restricted maximum likelihood method to determine *P* values for the effects of age and genotype. Asterisks indicate a significant difference between KO and the corresponding control (*P* < 0.05) after adjustment by the Bonferroni method. NS not significant.

To determine the effects of *Tafazzin*KO and *Crls1*KO, we created age-specific volcano plots. Deletion of *Tafazzin* caused few changes in the proteome, mostly among enzymes of the amino acid metabolism. In contrast, deletion of *Crls1* had a severe effect that strongly progressed with age. Most prominently, *Crls1*KO decreased the abundance of several mitochondrial proteins and increased the abundance of several enzymes of the amino acid metabolism in an age-dependent manner (Fig. 3A).

*Crls1*KO but not *Tafazzin*KO, prevented the postnatal increase in the abundance of respiratory enzymes, particularly during the second week of life when CRLS1 was almost fully ablated (Fig. 3B). Other mitochondrial proteins remained largely unaffected. For instance, the TOM complex, a constitutive protein of the outer membrane of mitochondria, did not change its tissue concentration with age nor was it altered by *Tafazzin*KO or *Crls1*KO (Fig. 3C). Both deletions did, however, increase the abundance of asparagine synthase (Fig. 3D), an enzyme of amino acid metabolism that is susceptible to stress (Lomelino et al, 2017). *Crls1*KO but not *Tafazzin*KO inhibited cardiac maturation (Fig. 3E) as demonstrated by the MYH6/MYH7 protein abundance ratio that reflects fetal-to-adult isoform switching of the myosin heavy chain (Guo and Pu, 2020).

**Figure 4:**
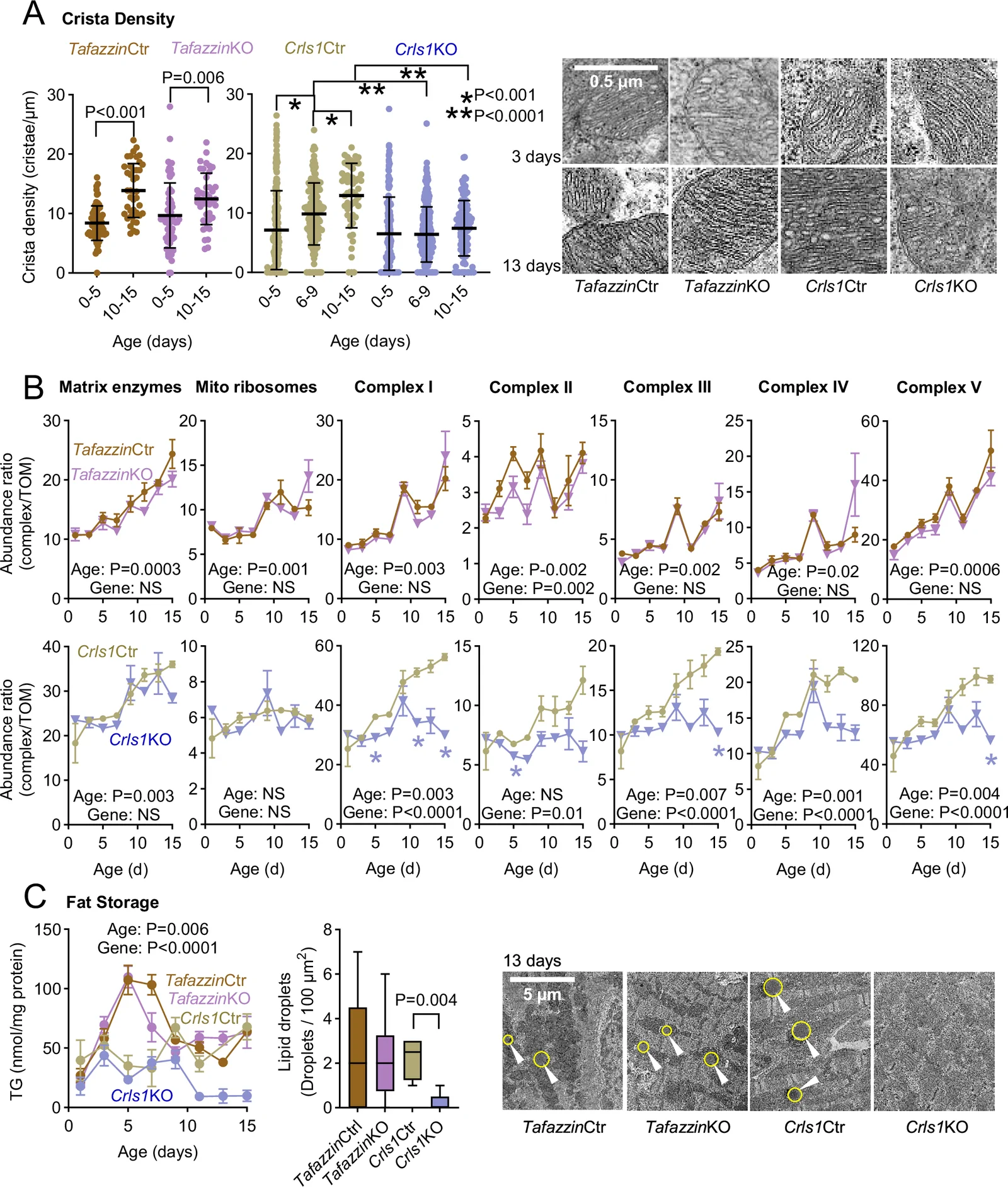
Effects of *Tafazzin*KO and *Crls1*KO on the postnatal maturation of cardiac mitochondria. (**A**) Heart tissue samples of neonatal mice were analyzed by electron microscopy at different ages. Three hearts were analyzed per genotype and age group. Each point represents a separate electron micrograph (42 ≤ *N* ≤ 313). Mean values are shown with standard deviations. Comparisons were made by unpaired, two-tailed *t* test. (**B**) Proteins were analyzed in heart homogenates. Data (means with standard deviations, *N* = 3 per age) are ratios of composite protein abundances. To calculate the abundance of a protein complex or a group of proteins, LFQ intensities of individual subunits (listed in Dataset EV2) were added. Mixed-effects analysis was performed using the restricted maximum likelihood method to determine *P* values for the effects of age and genotype. Asterisks indicate a significant difference between KO and the corresponding control (*P* < 0.05) after adjustment by the Bonferroni method. NS, not significant. (**C**) Triglyceride concentrations were measured in heart homogenates by LC–MS/MS at different ages (*N* = 3 per age). Mixed-effects analysis was performed using the restricted maximum likelihood method to determine *P* values for the effects of age and genotype. The density of lipid droplets was quantified by electron microscopy of 13-day-old mouse hearts (*N* = 4). Box‑and‑whisker plots indicate means, interquartile ranges, and “whiskers” extending to the data’s extremes. In electron micrographs, lipid droplets are marked by yellow circles and arrowheads. Comparisons between KOs and their respective controls were made by an unpaired, two-tailed *t* test. Source data are available online for this figure.

To follow the morphologic maturation of cardiac mitochondria, we collected serial images by electron microscopy. Normal mitochondria increased the density of cristae over the first 2 weeks of life, indicating that mitochondria ramped up their oxidative capacity. *Crls1*KO prevented the increase in crista density, whereas *Tafazzin*KO had no effect (Fig. 4A).

Next, we estimated the intramitochondrial concentration of respiratory complexes, ribosomes, and matrix enzymes. To this end, we added the label-free quantitation (LFQ) intensities of their subunits (specified in Dataset EV2) and divided the sum by the LFQ intensity of the TOM complex (sum of the TOM subunits, Dataset EV2). Since the abundance of TOM is an indicator of the total surface area of mitochondria, the ratio becomes a measure of intramitochondrial concentration.

We found that the concentration of respiratory complexes within mitochondria gradually increased during postnatal maturation. *Crls1*KO but not *Tafazzin*KO inhibited the accumulation of respiratory complexes within mitochondria. In contrast, neither deletion had any effect on the mitochondrial concentration of matrix proteins, such as matrix enzymes or ribosomal proteins (Fig. 4B). These results show that Crls1KO inhibited specifically the biogenesis of cristae by reducing the expansion of the inner membrane and by decreasing the expression of respiratory complexes.

Furthermore, *Crls1*KO dramatically decreased the concentration of triglycerides and the number of lipid droplets at the age of 2 weeks, consistent with severe deterioration of energy metabolism shortly before death. *Tafazzin*KO did not alter lipid droplets or triglycerides (Fig. 4C).

In summary, *Tafazzin*KO and *Crls1*KO mice were born with a normal cardiac proteome and with normal mitochondria. While *Tafazzin*KO hearts matured like wild-type, with mitochondria increasing the inner membrane surface and the intramitochondrial concentration of respiratory proteins, *Crls1*KO hearts failed to mature the internal structure and the composition of mitochondria.

### Biogenesis of the respiratory chain is dependent on the de novo synthesis of CL

Our data indicate a specific connection between de novo synthesis of CL and the formation of the respiratory chain. We discovered this connection in neonatal hearts, where postnatal maturation is associated with a dramatic increase in the intramitochondrial concentration of respiratory enzymes. *Crls1*KO prevented both the rise in the concentration of respiratory proteins within mitochondria and the expansion of the inner mitochondrial membrane. In contrast, *Tafazzin*KO had no obvious effect on the postnatal maturation of heart mitochondria despite lowering the tissue concentration of CL to a similar level. Thus, it is specifically the de novo synthesis of CL but not the remodeling of CL that asserts control over the biogenesis of cristae and the respiratory chain. This conclusion supports and expands previous claims that *Crls1* controls energy metabolism in brown adipose tissue and skeletal muscle (Sustarsic et al, 2018; Yoo et al, 2024). Our observation that *Crls1*KO reduces not only CL content but also CL remodeling is consistent with the idea that remodeling is dependent on assembly of the respiratory chain (Xu et al, 2019).

Although the two KOs were induced under the same promoter (*Myh6*), they may not have been equally effective at birth. Metabolic consequences of *Tafazzin*KO, such as altered species pattern and accumulation of lyso-CLs, were already prominent at birth, but the effects of *Crls1*KO developed gradually, as demonstrated by age-dependent loss of CRLS1 activity. We speculate that different half-lives of TAFAZZIN and CRLS1 are responsible for this discrepancy. TAFAZZIN has a very short half-life in mammalian cell cultures (Xu et al, 2015), whereas most other proteins of the inner mitochondrial membrane are known for their longevity (Bomba-Warczak et al, 2021; Krishna et al, 2021).

Surprisingly, the lack of CL could not explain the phenotypic difference between *Crls1*KO and *Tafazzin*KO. Both strains were born with near normal concentrations of total CL and developed a deficiency gradually in the postnatal period by failing to increase the CL concentration. Although CL became slightly more concentrated in *Tafazzin*KO than in *Crls1*KO by the end of the second week, the difference was not significant (*P* > 0.2). However, CL deficiencies were caused by different mechanisms in the two KO models. In *Crls1*KO, insufficient synthesis reduced CL and at the same time increased the concentration of CL precursors, such as PG. In *Tafazzin*KO, accelerated degradation reduced CL and increased the concentration of partially degraded CLs, such as MLCL and DLCL. Potentially, these metabolites are more important for the phenotype than CL itself. For instance, the rise of CL precursors may downregulate the expression, import, or assembly of respiratory proteins. MLCL, on the other hand, may substitute for CL to some extent and therefore partially support mitochondrial function. Further studies are needed to test these speculations, but the present data do suggest that the regulatory effect of Crls1 on the respiratory chain is independent of the CL concentration.

Differences exist between mouse and human cardiomyocytes, most notably in the timing of the proliferation-to-hypertrophy transition and in the number of nuclei per cell (Guo and Pu, 2020), which may limit the human relevance of our data. Patients with *Tafazzin* mutations (Barth syndrome) often develop cardiomyopathy in infancy (Kang et al, 2016; Roberts et al, 2012), whereas TAFAZZIN-deficient mice develop cardiomyopathy only after weaning regardless of whether *Tafazzin* is deleted in the whole body or only in cardiomyocytes (Wang et al, 2020; Zhu et al, 2021).

In conclusion, deletion of *Crls1* (enzyme of CL de novo synthesis) from cardiomyocytes causes progressive heart failure and early death in newborn mice. Cardiomyocyte-restricted deletion of *Tafazzin* (enzyme of CL remodeling), on the other hand, does not affect cardiac function until mice reach adulthood (Wang et al, 2020; Zhu et al, 2021). We show that de novo synthesis of CL but not concentration of CL, strongly affects biogenesis of the respiratory chain, controlling both the expansion of the inner membrane and the mitochondrial expression of respiratory proteins.

## Methods

**Table:** Reagents and tools table

Reagent/resource	Reference or source	Identifier or catalog number
**Experimental models**		
C57BL/6N-A^tm1Brd^ Crls1^tm1a(EUCOMM)Wtsi^/WtsiOulu (*Mus musculus*)	European Mouse Mutant Archive	EM:10865
B6.129S4-Gt(ROSA)26Sortm2(FLP*)Sor/J (*Mus musculus*)	The Jackson Laboratory	Strain 012930
B6.FVB-Tg[Myh6-cre]2182Mds/J (*Mus musculus*)	The Jackson Laboratory	Strain 011038
Tafazzin^flox/flox^ (*Mus musculus*)	Ren et al (2019)	N/A
**Chemicals, enzymes, and other reagents**		
Neonatal Heart Dissociation Kit	Miltenyi Biotec	130-098-373
Neonatal Cardiomyocyte Isolation Kit	Miltenyi Biotec	130-100-825
CDP-dioleoylglycerol	Avanti	870520
dimyristoyl-phosphatidylglycerol	Avanti	840445
Cardiolipin Standard Mix I	Avanti	LM6003
Mouse SPLASH	Avanti	330710
Ultra C18 reversed-phase column	Restek	9174312
Trypsin Gold	Promega	V5280
**Software**		
Xcalibur, Version 4.1.50	Thermo Fisher Scientific	N/A
LipidSearch, Version 5.1.8	Thermo Fisher Scientific	N/A
MaxQuant, Version 2.5.1.0	Max Planck Institute of Biochemistry	N/A
**Other**		
Vevo 770 Ultrasound machine	Visual Sonics	N/A
QExactive HF-X mass spectrometer	Thermo Fisher Scientific	N/A
QExactive HF mass spectrometer	Thermo Fisher Scientific	N/A
Vanquish UHPLC	Thermo Fisher Scientific	N/A

### Mice

All protocols were approved by the Institutional Animal Care and Use Committee of the NYU School of Medicine and conform to the Guide for the Care and Use of Laboratory Animals published by the National Institutes of Health (NIH). Protocol # 160406. Mice were housed under temperature-controlled conditions under a 12-h light/dark cycle with free access to drinking water and food.

Mice with floxed cardiolipin synthase gene (*Crls1*^flox/flox^) were generated by crossing the conditionally ready strain (C57BL/6N-A^tm1Brd^ Crls1^tm1a(EUCOMM)Wtsi^/WtsiOulu) from the European Mouse Mutant Archive with the FLPo deleter strain (B6.129S4-*Gt(ROSA)26Sor*^tm2(FLP*)Sor^/J, Strain # 012930) from The Jackson Laboratory to remove the reporter-tagged insertion. Crls1^flox/flox^ mice were crossed with *αMyHC-cre* mice (B6.FVB-Tg[Myh6-cre]2182Mds/J, Strain # 011038) from The Jackson Laboratory, to generate *αMyHC-cre;Crls1*^*flox*/+^ mice. Cardiomyocyte-specific *Crls1*KO mice (*αMyHC-cre*; Crls1^flox/flox^) were generated by crossing Crls1^flox/flox^ mice with *αMyHC-cre;Crls1*^*fl*ox/+^ mice. Genotyping was performed by polymerase chain reaction in tail clips. To generate cardiomyocyte-specific *Tafazzin*KO mice (*αMyHC-cre*; Tafazzin^flox/Y^), female Tafazzin^flox/flox^ (Ren et al, 2019) were crossed with male *αMyHC-cre* mice.

### Heart specimens

Hearts were harvested from mice euthanized by CO_2_ narcosis/asphyxiation. Hearts were excised through a vertical anterior thoracotomy, trimmed from attached non-cardiac tissue, rinsed in cold phosphate-buffered saline, and either homogenized in water or processed for the purification of cardiomyocytes. In order to purify cardiomyocytes, hearts were dissociated into single-cell suspensions using the Neonatal Heart Dissociation kit from Miltenyi Biotec, which applies a combination of enzymatic degradation and mechanical disruption. Non-cardiomyocytes were removed from the cell suspension by affinity binding to monoclonal antibodies. For that purpose, we used the Neonatal Cardiomyocyte Isolation kit from Miltenyi Biotec. Heart and cardiomyocyte samples were stored at −80 °C. The protein concentration was determined by the method of Lowry (Lowry et al, 1951).

### Echocardiography

Transthoracic echocardiography was performed on anesthetized mice as previously described (Phoon et al, 2012; Phoon et al, 2024). In brief, mice aged 5 days and older were anesthetized with isoflurane via nose cone (induction: 2.5% isoflurane; maintenance: 1% isoflurane at a flow rate of 1 l/min) with strict thermoregulation (37 ± 0.5 °C) to optimize physiological conditions and reduce hemodynamic variability. Because depilation of the chest was not necessary in younger mice, they were gently held and quickly scanned ( < 1 min) without anesthesia. Two-dimensional echocardiography of the parasternal short axis view was performed using the Vevo 770 (Visual Sonics, Toronto, ON, Canada) with the RMV-704, 40 MHz center frequency transducer (age >4 days) or the RMV-711, 55 MHz center frequency transducer (age <4 days). To minimize bias, we performed all echocardiographic measurements offline while blinded to the genotype.

### Microscopy

Paraffin sections of 6 µm thickness were prepared for histological evaluations of the hearts. The sections were stained with hematoxylin and eosin. For electron microscopy, heart tissue samples (< 3 mm diameter) were fixed in glutaraldehyde and osmium tetroxide. Fixed samples were stained with uranyl acetate. The buffer was gradually exchanged with ethanol, followed by resin exchange and heat polymerization. Sections of 50–100 nm were cut with a Leica Ultracut UCT microtome and collected on grids. Sections were stained with uranyl acetate and Sato Lead stains and imaged with a Philips CM12 transmission electron microscope. Random images were collected at different magnifications. Crista densities were determined at a magnification of 5000. Crista density was measured as the number of cristae that intersect a line drawn across the mitochondrion roughly perpendicular to the main crista orientation. Morphometric data were collected by a blinded observer in randomly selected images.

### CRLS1 activity assay

Heart homogenate, corresponding to 70 µg protein, was incubated for 45 min at 37 °C in 0.1 mL buffer containing 10 mM Tris (pH 8.0), 5 mM CoCl_2_, 0.1 mM dimyristoyl-phosphatidylglycerol (PG14:0/14:0), and 0.1 mM CDP-dioleoylglycerol (CDPDG18:1/18:1). Reactions were stopped by the addition of 2 mL methanol and 1 mL chloroform. Cardiolipin standard mix I (Avanti Polar Lipids), containing 101 pmol CL14:1/14:1/14:1/15:1, 92.2 pmol CL15:0/15:0/15:0/16:1, 84.7 pmol CL14:1/22:1/22:1/22:1, and 77.1 pmol CL14:1/24:1/24:1/24:1, was added. Lipids were extracted (Bligh and Dyer, 1959), dried under nitrogen, and re-suspended into 0.1 mL chloroform/methanol 1:1. An aliquot of 5 µL was applied to LC–MS/MS analysis as described below. To determine the amount of CL14:0/14:0/18:1/18:1 formed during the incubation, signal intensities of CL14:0/14:0/18:1/18:1 and of 2 CL standards (CL14:1/14:1/14:1/15:1, CL15:0/15:0/15:0/16:1) were quantified in the Qual Browser of Xcalibur (Thermo Fisher Scientific, West Palm Beach, FL 33407, Version 4.1.50). We have shown that the concentration of CL14:0/14:0/18:1/18:1 rises in a linear relation to the incubation time and to the amount of heart homogenate. CRLS1 activities were expressed as fmol CL14:0/14:0/18:1/18:1 formed per minute and µg protein.

### Lipidomics

Heart homogenates or cardiomyocytes, containing about 1 mg protein, were extracted into chloroform/methanol as described (Bligh and Dyer, 1959). To this end, samples were homogenized in 0.2 mL water, suspended in 3 ml methanol/chloroform (2:1), and incubated at 37 °C for 30 min to denature proteins. A mixture of internal standards was added consisting of Cardiolipin standard mix I (303 pmol CL14:1/14:1/14:1/15:1, 277 pmol CL15:0/15:0/15:0/16:1, 254 pmol CL14:1/22:1/22:1/22:1, 231 pmol CL14:1/24:1/24:1/24:1) and Mouse SPLASH (625 pmol PC15:0/18:1d7, 44 pmol PE15:0/18:1d7, 125 pmol PS15:0/18:1d7, 31 pmol PG15:0/18:1d7, 125 pmol PI15:0/18:1d7, 62.5 pmol PA15:0/18:1d7, 281 pmol LPC18:1d7, 12.5 pmol LPE18:1d7, 1562 pmol cholesterol ester18:1d7, 125 pmol plasmenyl-PC18:0/18:1d9, 94 pmol DG15:0/18:1d7, 219 pmol TG15:0/18:1d7/15:0, 125 pmol SMd18:1/18:1d9, 31 pmol plasmenyl-PE18:0/18:1d9). Standards were obtained from Avanti Polar Lipids. Chloroform and water were added (1 ml each), the samples were vortexed, and phase separation was achieved by centrifugation. The lower phase was collected, dried under nitrogen, and re-dissolved in 0.2 ml chloroform/methanol (1:1). Lipids were analyzed by LC-ESI-MS/MS on a QExactive HF-X instrument coupled directly to a Vanquish UHPLC (Thermo Fisher Scientific, Waltham, MA, USA). An aliquot of 5 µl was injected into a Restek Ultra C18 reversed-phase column (Restek Corporation, Bellefonte, PA, USA; 100 × 2.1 mm; particle size 3 µm) that was kept at a temperature of 50 °C. Chromatography was performed with solvents A and B at a flow rate of 0.15 mL/min. Solvent A contained 600 ml acetonitrile, 399 ml water, 1 ml formic acid, and 0.631 g ammonium formate. Solvent B contained 900 ml 2-propanol, 99 ml acetonitrile, 1 ml formic acid, and 0.631 g ammonium formate. The chromatographic run time was 40 min, changing the proportion of solvent B in a non-linear gradient from 30 to 35% (0–2 min), from 35 to 67% (2–5 min), from 67 to 83% (5–8 min), from 83 to 91% (8–11 min), from 91 to 95% (11–14 min), from 95 to 97% (14–17 min), from 97 to 98% (17–20 min), from 98 to 100% (20–25 min), and from 100 to 30% (25–26 min). For the remainder of the run time, the proportion of solvent B stayed at 30% (26–40 min). To analyze acyl-carnitines, cholesterol esters, and glycerides, the mass spectrometer was operated in positive ion mode. For all other lipids, it was operated in negative ion mode. The spray voltage was set to 4 kV, and the capillary temperature was set to 350 °C. MS1 scans were acquired in profile mode at a resolution of 120,000, an AGC target of 1e6, a maximal injection time of 65 ms, and a scan range of 200–2000 *m/z*. MS2 scans were acquired in profile mode at a resolution of 30,000, an AGC target of 3e6, a maximal injection time of 75 ms, a loop count of 7, and an isolation window of 1.7 *m/z*. The normalized collision energy was set to 30 and the dynamic exclusion time to 31 s. For lipid identification and quantitation, data were analyzed by the software LipidSearch 5.1.8 (Thermo Fisher Scientific, Waltham, MA, USA). The general database was searched with a precursor tolerance of 2 ppm, a product tolerance of 0.01 Da, and a product intensity threshold of 1.0%.

### Proteomics

Protein abundances in heart homogenates were determined by label-free relative quantitative proteomics (MaxLFQ) using data-independent acquisition (MaxDIA) (Sinitcyn et al, 2021). Proteins were purified and concentrated by a brief SDS-PAGE run designed to focus all proteins into a single band of about 1 cm × 0.5 cm. Gels were washed three times in double-distilled water for 15 min each. Proteins were visualized and fixed by staining with EZ-Run Protein staining solution (ThermoFisher Scientific, USA). The stained protein gel regions were excised and de-stained three times in 50% methanol for 15 min each time. Then the gel regions were treated with 50 mM NH_4_HCO_3_ in 30% acetonitrile until completely de-stained. Finally, each gel sample was dehydrated with 100% acetonitrile. Cysteinylated proteins were reduced with 10 mM dithiothreitol and alkylated with 55 mM iodoacetamide. After two washes with 50 mM NH_4_HCO_3_ in 30% acetonitrile, samples were dried by vacuum centrifugation. Dried gel samples were digested overnight with mass spectrometry grade trypsin (Trypsin Gold, Promega, Madison, WI, USA) at 5 ng/µl in 50 mM NH_4_HCO_3_ digest buffer. After acidification with 10% formic acid, peptides were extracted with 5% formic acid/50% Acetonitrile (v/v) and concentrated to a small droplet using vacuum centrifugation. Desalting of peptides was done using hand-packed SPE Empore C18 Extraction Disks as described (Rappsilber et al, 2007). Desalted peptides were again concentrated and reconstituted in 10 µL 0.1% formic acid in water. Aliquots of the peptides were analyzed by nano-liquid chromatography followed by tandem mass spectrometry (nano-LC–MS/MS) using an Easy nLC 1000 equipped with a self-packed 75 µm × 20 cm reverse phase column (ReproSil-Pur C18, 3 µm, Dr. Maisch GmbH, Germany) coupled online to a QExactive HF Orbitrap mass spectrometer via a Nanospray Flex source (all instruments from Thermo Fisher Scientific, Waltham, MA, USA). Analytical column temperature was maintained at 50 °C by a column oven (Sonation GmBH, Germany). Peptides were eluted with a 3–40% acetonitrile gradient over 110 min at a flow rate of 250 nL/min. The mass spectrometer was operated in data-independent-analysis mode and profile mode with survey scans acquired at a resolution of 120,000 (at *m/z* 200) over a scan range of 300–1650 *m/z*. Following the survey scans, 30 groups of precursors were selected for fragmentation with sliding isolation windows to include peptide *m/z* values ranging from 364 to 1370 Th. The default maximum charge state was set to 4, and resolution was set to 30,000. In MS/MS, the fixed first mass was set to 200 Da. The normalized collision energy was 27. The maximum injection times for the survey were set to 60 ms, and the maximum injection time of MS/MS scans was set to auto. The ion target value for both scan modes was set to 3e6. Data were analyzed by the software MaxQuant (version 2.5.1.0), with the option for MaxDIA analysis (Sinitcyn et al, 2021). To identify peptides, we used mouse in silico-generated spectral libraries that were created from the Uniprot mouse protein sequence database (downloaded on 06/18/2020; 21,989 entries). The library was obtained from the Max Planck Institute of Biochemistry Data share drive (MPIB Data share https://datashare.biochem.mpg.de/s/qe1IqcKbz2j2Ruf?path=%2FDiscoveryLibraries). To determine the relative abundance of a protein across a set of samples, we used its label-free quantification (MaxLFQ) intensity calculated by MaxDIA (Sinitcyn et al, 2021), which normalizes the protein intensity by normalizing peptide intensities (minimum peptide ratio count=2) across samples. To determine the relative abundance of a protein complex, we summed LFQ intensities of all observed subunits.

### Statistical analysis

Data are biological replicas collected at multiple neonatal ages (1–15 days). Samples were analyzed in two separate runs, one for the *Tafazzin*Ctrl and *Tafazzin*KO pair and another for the *Crls1*Ctr and *Crls1*KO pair. Data were analyzed by the software GraphPad Prism 10. Comparisons between groups were made by *t* test (unpaired, two-tailed). The proportion of genotypes at different ages was analyzed by a binomial test (two-tailed). To evaluate age-dependent progression of variables in different genotypes, mixed-effects analysis was performed by the restricted maximum likelihood method, applying a full model with interactive effects and Geisser-Greenhouse correction (default parameters of the GraphPad Prism 10). Genotypes were compared at each age by the Bonferroni method. Significance threshold of adjusted *P* values was set to 0.05. In volcano plots, the significance level was determined by the Benjamini–Hochberg procedure using a false discovery rate of 0.05. The significance threshold was 10^−1.99^ for lipidomics data and 10^−2.17^ for proteomics data. Consequently, *P *< 0.01 was considered significant in lipidomics volcano plots, and *P* < 0.001 was considered significant in proteomics volcano plots.

## Supplementary information

Peer Review File

Dataset EV1

Dataset EV2

Source data Fig. 1

Source data Fig. 4

Expanded View Figures

## Data Availability

Proteomics LC–MS data (proteomics raw mass spectrometry data, peak lists, and results) that support the findings of this study are deposited to the ProteomeXchange Consortium via the MassIVE partner repository and can be retrieved with the accession code MSV000099296 and with the dataset identifier PXD06888. The source data of this paper are collected in the following database record: biostudies:S-SCDT-10_1038-S44319-026-00864-8.
