# Supplementary material for: De novo synthesis of cardiolipin controls respiratory chain biogenesis in neonatal mouse hearts
Source: EMBO Rep. 2026 Jul 7;27(16):4653–67. doi: 10.1038/s44319-026-00864-8 (PMC13503872; doi:10.1038/s44319-026-00864-8)
Supplement: Supplementary file 1 [file 44319_2026_864_MOESM1_ESM.pdf]

EMBO reports

De novo synthesis of cardiolipin controls respiratory chain biogenesis in neonatal mouse hearts

Mindong Ren, Yang Xu, Colin Phoon, Hediye Erdjument-Bromage, Thomas Neubert, and Michael Schlame

Corresponding author(s): Mindong Ren (mindong.ren@nyulangone.org) , Michael Schlame (michael.schlame@med.nyu.edu)

Review Timeline:

Submission Date:	21st Nov 25
Editorial Decision:	9th Jan 26
Revision Received:	2nd Mar 26
Editorial Decision:	11th Jun 26
Revision Received:	15th Jun 26
Accepted:	25th Jun 26

Editor: Martina Rembold

Transaction Report:

(Note: With the exception of the correction of typographical or spelling errors that could be a source of ambiguity, letters and reports are not edited. Depending on transfer agreements, referee reports obtained elsewhere may or may not be included in this compilation. Referee reports are anonymous unless the Referee chooses to sign their reports.)

Dear Dr. Schlame

Thank you for the submission of your manuscript to EMBO reports. I apologize for the delay in handling it, but we have now received the full set of referee reports as well as referee cross-comments that are all pasted below.

As you will see, the referees acknowledge that the findings are potentially interesting, but they also raise a number of concerns on your data. Referee #1 and #3 both comment on the complexome analysis shown in Figure 5 and have similar concerns regarding the interpretation of these data, i.e., that co-migration does not demonstrate direct binding. Referee #3 suggests removing these preliminary data and I tend to agree with this suggestion, unless further data can be provided. Moreover, Referee #3 suggests broadening the analysis to female mice. This would indeed strengthen the relevance but appears to require a lot of additional work and is therefore not mandatory. That said, Figure 1A suggests this you might have analysed female mice and if so, these data should be added. All other concerns need to be addressed.

I would thus like to invite you to revise your manuscript with the understanding that the referee concerns must be fully addressed and their suggestions taken on board. Please address all referee concerns in a complete point-by-point response. Acceptance of the manuscript will depend on a positive outcome of a second round of review. It is EMBO reports policy to allow a single round of major revision only and acceptance or rejection of the manuscript will therefore depend on the completeness of your responses included in the next, final version of the manuscript.

We realize that it is difficult to revise to a specific deadline. In the interest of protecting the conceptual advance provided by the work, we recommend a revision within 3 months (11th April 2026). Please discuss the revision progress ahead of this time with the editor if you require more time to complete the revisions.

You can either publish the study as a short report or as a full article. For short reports, the revised manuscript should not exceed 27,000 characters (including spaces but excluding materials & methods and references) and 5 main plus 5 expanded view figures. The results and discussion sections must further be combined, which will help to shorten the manuscript text by eliminating some redundancy that is inevitable when discussing the same experiments twice. For a normal article there are no length limitations, but it should have more than 5 main figures and the results and discussion sections must be separate. In both cases, the entire materials and methods must be included in the main manuscript file.

IMPORTANT NOTE: we perform an initial quality control of all revised manuscripts before re-review. Your manuscript will FAIL this control and the handling will be DELAYED if the following APPLIES:
1) A data availability section providing access to data deposited in public databases is missing. If you have not deposited any data, please add a sentence to the data availability section that explains that.
2) Your manuscript contains statistics and error bars based on n=2. Please use scatter blots in these cases. No statistics should be calculated if n=2.

When submitting your revised manuscript, please review the submission guidelines (https://link.springer.com/journal/44319/submission-guidelines#cms-Revised-submissions) and carefully review the instructions that follow below. Failure to include requested items will delay the evaluation of your revision.

1) a .docx formatted version of the manuscript text (including legends for main figures, EV figures and tables). Please make sure that the changes are highlighted to be clearly visible.

2) individual production quality figure files as .eps, .tif, .jpg (one file per figure). See hhttps://media.springernature.com/original/springer-cms/rest/v1/content/27825798/data/v1 for more info on how to prepare your figures.

3) We replaced Supplementary Information with Expanded View (EV) Figures and Tables that are collapsible/expandable online. A maximum of 5 EV Figures can be typeset. EV Figures should be cited as 'Figure EV1, Figure EV2" etc... in the text and their respective legends should be included in the main text after the legends of regular figures.

- For the figures that you do NOT wish to display as Expanded View figures, they should be bundled together with their legends in a single PDF file called *Appendix*, which should start with a short Table of Content. Appendix figures should be referred to in the main text as: "Appendix Figure S1, Appendix Figure S2" etc. See 'Expanded View' section of the submission guidelines.

- Additional Tables/Datasets should be labeled and referred to as Table EV1, Dataset EV1, etc. Legends have to be provided in a separate tab in case of .xls files. Alternatively, the legend can be supplied as a separate text file (README) and zipped together with the Table/Dataset file.

4) a .docx formatted letter INCLUDING the reviewers' reports and your detailed point-by-point responses to their comments. As part of the EMBO Press transparent editorial process, the point-by-point response is part of the Review Process File (RPF),

which will be published alongside your paper.

5) a complete author checklist, which you can download from our author guidelines. Please insert information in the checklist that is also reflected in the manuscript. The completed author checklist will also be part of the RPF.

6) Please note that all corresponding authors are required to supply an ORCID ID for their name upon submission of a revised manuscript (<https://orcid.org/>), which can be linked to your profile in the manuscript submission system.

7) Before submitting your revision, primary datasets produced in this study need to be deposited in an appropriate public database. Please remember to provide a reviewer password if the datasets are not yet public. The accession numbers and database should be listed in a formal "Data Availability" section placed after Methods. Please note that the Data Availability Section is restricted to new primary data that are part of this study. * Note - All links should resolve to a page where the data can be accessed. *

If your study has not produced novel datasets, please mention this fact in the Data Availability Section.

8) At EMBO Press we ask authors to provide source data for the main manuscript figures. You will receive a separate email with instructions for providing source data with your revised manuscript, including how to upload and organize the files.

9) Our journal also encourages inclusion of *data citations in the reference list* to directly cite datasets that were re-used and obtained from public databases. Data citations in the article text are distinct from normal bibliographical citations and should directly link to the database records from which the data can be accessed. In the main text, data citations are formatted as follows: "Data ref: Smith et al, 2001" or "Data ref: NCBI Sequence Read Archive PRJNA342805, 2017". In the Reference list, data citations must be labeled with "[DATASET]". A data reference must provide the database name, accession number/identifiers and a resolvable link to the landing page from which the data can be accessed at the end of the reference.

10) Regarding data quantification, the following points must be specified in each figure legend:

- the name of the statistical test used to generate error bars and P values,

- the number (n) of independent experiments (please specify technical or biological replicates) underlying each data point,

- the nature of the bars and error bars (s.d., s.e.m.),

- If the data are obtained from n Program fragment delivered error ``Can't locate object method "less" via package "than" (perhaps you forgot to load "than"?) at //ejpvfs23/sites23b/embor_www/letters/embor_decision_revise_and_review.txt line 54.' 2, use scatter blots showing the individual data points.

Discussion of statistical methodology can be reported in the materials and methods section, but figure legends should contain a basic description of n, P and the test applied.

- Please also include scale bars in all microscopy images.

11) The journal requires a statement specifying whether or not authors have competing interests (defined as all potential or actual interests that could be perceived to influence the presentation or interpretation of an article). In case of competing interests, this must be specified in your disclosure statement.

12) All Materials and Methods need to be described in the main text using our 'Structured Methods' format, which is required for all research articles. According to this format, the Methods section includes a Reagents and Tools Table (listing key reagents, experimental models, software and relevant equipment and including their sources and relevant identifiers) followed by a Methods and Protocols section describing the methods using a step-by-step protocol format. The aim is to facilitate adoption of the methodologies across labs. More information on how to adhere to this format as well as a downloadable template (.docx) for the Reagents and Tools Table can be found in our author guidelines.

We would also welcome the submission of cover suggestions, or motifs to be used by our Graphics Illustrator in designing a cover.

As part of the EMBO publication's Transparent Editorial Process, EMBO reports publishes online a Review Process File (RPF) to accompany accepted manuscripts. This File will be published in conjunction with your paper and will include the referee reports, your point-by-point response and all pertinent correspondence relating to the manuscript.

You are able to opt out of this by letting the editorial office know (emboreports@embo.org). If you do opt out, the Review Process File link will point to the following statement: "No Review Process File is available with this article, as the authors have chosen not to make the review process public in this case."

I look forward to seeing a revised form of your manuscript when it is ready.

Yours sincerely,

Martina Rembold, PhD
Senior Editor
EMBO reports

==================

Referee #1:

Ren et al. present a very interesting study showing that cardiomyocyte-restricted deletion of Crls1 (CL synthase) but not Tafazzin (remodeling) disrupts postnatal cardiac maturation, preventing the normal rise in cristae density and intra-mitochondrial concentration of respiratory proteins. Consequently, animals develop heart failure and died by ~P16. Conversely, Tafazzin loss reduces CL remodeling but does not block early postnatal maturation. The authors propose a mechanistic explanation: TAFAZZIN and ABHD18 (a CL lipase) co-migrate with nascent complex I assemblies (assessed by complexome profiling of P13 neonatal heart mitochondria) whereas de novo biosynthetic enzymes (PGS1/TAMM41/PTPMT1/CRLS1) do not-suggesting remodeling enzymes transiently associate with complex I precursors.
Those conclusions are based on lipidomic and proteomic analysis together with cardiac function studies and electron microscopy
analysis of the cardiac mitochondria. The lipidomic studies performed in 2-day intervals across the first 2 postnatal weeks showed that:
CL rises in controls but not in either KO.
MLCL/DLCL accumulate in Tafazzin_KO, whereas PG accumulates in Crls1_KO.
CL species become longer/more unsaturated with age in WT; this maturation is blocked or slowed in the KOs.
The proteomics analysis shows:
A strong age-dependent increase of OXPHOS complexes, while TOM complex remains stable, in WT. This is abrogated in Crls1_KO (but not in Tafazzin_KO)
ASNS increases in both KOs.
The Cardiac function study found:
Dilated cardiomyopathy in Crls1_KO, but not in Tafazzin_KO during neonatal period.
The EM analysis revealed an increase in cristae density during weeks 1-2 in WT and Tafazzin_KO but not in Crls1_KO. It also showed a sharp drop in lipid droplets/TG in Crls1_KO.
The work is concise yet data-rich, with clear physiological relevance and a mechanistic angle on membrane biogenesis/respiratory chain assembly. The side-by-side comparison of de novo synthesis vs remodeling in postnatal heart maturation is timely and conceptually novel. The finding that CL synthesis (CRLS1)-but not remodeling-controls the age-dependent rise of respiratory protein concentration and cristae expansion in vivo is mechanistically intriguing and of broad interest to mitochondrial biology and developmental physiology. However, it may need targeted additional evidence and improvements in statistical reporting and figure clarity to be fully convincing.

Major points:

1. Statistics and multiple testing across omics are required: The Volcano plots use $p<0.01$ with a 4-fold cutoff but there's no multiple-testing correction stated for hundreds to thousands of features. Please report adjusted q-values for all lipid and protein differential analyses; ensure effect sizes and confidence intervals accompany bar/line plots. With respect to the age × genotype designs sampled longitudinally (2-day intervals), a linear mixed-effects model (or at minimum two-way ANOVA with interaction and proper post-hoc FDR correction) is more appropriate than multiple unpaired t-tests. Clarify the exact model and assumptions (normality/variance).
2. The statement: "associate with nascent complex I" is not fully supported by the data that only support that "co-migrate with complex I assembly intermediates". This shouls be correct throughout the text unless additional evidence is provided The co-migration of TAFAZZIN/ABHD18 with complex I precursors is suggestive but not yet definitive evidence for physical interaction or functional coupling; this is correlative (complexome profiling). The language should be tempered or supported by orthogonal approaches. Complexome profiling shows co-migration, not binding. To consolidate this claim additional independent experimental confirmation is needed. Examples of those approaches may be Proximity labeling (APEX2/BioID) of complex I assembly intermediates to capture TAZ/ABHD18 enrichment. Crosslinking-MS or co-IP from digitonin-solubilized mitochondria under conditions that preserve the assembly intermediates. Replicate complexomes of the Tafazzin_KO and or ABHD18_KO showing that the mobility and/or the amount of the proposed CI interacting partners is affected when compared with control and Crls1_KO complexomes.
3. Survival and echocardiography presentation
Figure 1A: Replace genotype frequency vs age with a Kaplan-Meier survival curve (include N per group, events, censoring, log-rank p-value, and hazard ratio with 95% CI). The text states ~16-day mean lifespan for Crls1_KO-this belongs in a formal survival analysis.
Figure 1F (echo): Show representative M-mode images and provide full echo parameters (LVEDD, LVESD, LVFS, EF if

measured, heart rate under anesthesia, temperature), plus n and exact p-values. The methods are solid and blinded, but readers must see the data.
4. Sex as a biological variable. The authors state analyses were done only in males because Tafazzin is X-linked, yet Figure 1A labels "Crls1 (male & female)." Please clarify and harmonize the sex used per genotype across all experiments. Thus provide a brief analysis showing no sex effect in Crls1_KO or restrict to males consistently.
5. Clarify sampling and material used for each assay. Specify, for each figure, whether lipids/proteins were measured in whole heart homogenate, purified mitochondria, or isolated cardiomyocytes (only CL at P3 cardiomyocytes is explicitly stated). The lipidomics/proteomics/EM sections should clearly map to tissue fraction and age.
6. Causal link between CRLS1 loss and suppressed OXPHOS within mitochondria. The authors infer reduced intra-mitochondrial concentrations of OXPHOS from LFQ/TOM normalization. Consider corroborating with: BNPAGE quantification of complexes and supercomplexes; enzymatic activities (CI-CV) and oxygen consumption in permeabilized fibers or isolated cardiomyocytes; or mitochondrial mass markers (mtDNA copy number, citrate synthase) to disentangle content vs concentration changes.
7. Interpreting lipidomic differences mechanistically need to be tempered. The PG accumulation (Crls1_KO) vs MLCL/DLCL accumulation (Tafazzin_KO) is compelling. But the solid mechanistic interpretation may require to be strengthened by testing whether PG elevation per se can depress OXPHOS expression/assembly (e.g., PG supplementation in cardiomyocytes or genetic PGS1 perturbation), or by rescue with CRLS1 re-expression in Crls1_KO background (even partial, AAV if feasible). I am not thinking that these experiments are required for this manuscript, but the claim should be tempered.
8. CRLS1 activity assay validation. The assay uses PG14:0/14:0 + CDPDG18:1/18:1 to form CL14:0/14:0/18:1/18:1, a non-endogenous product-as you note in Figure S1. It would be convenient to add a standard curve and LOD/LOQ and specificity controls (e.g., heat-inactivation; metal chelation).
9. Figures need to be improved for clarity and better understanding.
Figure 1 (phenotypes & survival/echo) required legend refinement: Explicitly define what each panel represents. State n per time point, statistical test, two-tailed, and report exact p. Add Kaplan-Meier as noted; include representative M-mode frames and scale bars on histology.
Figure 2 (lipidomics): For volcano plots, display FDR-adjusted significance ($q<0.05$) and label key features (PG, CL, MLCL, DLCL). Add a methods inset clarifying sample source (whole heart vs mitochondria). In species plots and unsaturation/chain-length trajectories, include n per age, CIs, and exact p-values.
Figure 3 (proteomics): Clearly indicate how "OXPHOS/TOM" ratios were computed (sum of LFQs for subunits listed in Data S2?). Consider adding a heatmap of complex I/III/IV/V subunits over age for each genotype (z-scored per protein), with FDR. Include rationale for MYH6/MYH7 ratio as maturation marker and consider additional maturation markers more related with OXPHOS like the expression of paralogue genes for Complex IV related with cardiomyocyte maturation.
Figure 4 (EM and droplets/TG): Show representative EM images for each genotype and age (multiple magnifications), with quantification overlay. Provide blinding statement for EM counting. Ensure lipid droplet density is measured across comparable tissue regions.
Figure 5 (complexome): Provide replicate overlays of TAZ/ABHD18 peaks with ECSIT/TMEM126B/TMEM186 to visualize co-migration; add mass ladder ticks to the x-axis across all panels for ease of interpretation. Clarify digitonin ratio (6 g/g) rationale and whether alternate detergents were tested.
Supplementary Figures S1-S2
S1 (CRLS1 assay): add time course and protein titration plots with linear fits and r^2, plus the controls suggested above.
S2 (age-dependent CL species): enrich with stacked barplots or ridge plots across ages; indicate N=3 in the panel and include exact species lists in Data S1 for reproducibility.

Referee #2:

In the manuscript by Ren et al. the authors describe how cardiolipin is affecting cardiomyocyte maturation in the mammalian heart. It is a novel study that is well described and the authors made good graphical illustrations of their findings. I only have minor but important comments to address in the manuscript:

In figure 1, the authors show the mouse hearts in the different genetypes. It seems that the KO mice have smaller hearts than the control groups. Was the heart tissue weighed? And if yes, was there any differences?

It is not fully clear how many mice there were in each group in the analyses performed. In figure 2 legend, it is mentioned that 6 biological replicas per genotype and age group were used, however for some of the omics performed i.e. figure 3 B-E only N=3, but also for the lipidomics etc? Why was not all 6 mice used for the omics to strengthen the statistics?

Another comment to the data analysis is how well represented the respiratory chain proteins are represented in the proteomics. This should be clear in the legends of the figures i.e. figure 3, 4 and 5.

For the discussion, it would be relevant to discuss the differences between human and mouse cardiomyocyte maturation and compare this to their own findings. Also, the findings that Tafazzin variants does not lead to affect cardiac function until the mice reaches adulthood, whereas the human disease often manifests during early childhood? The authors should discuss this in the manuscript.

Referee #3:

Ren et al. investigated how postnatal maturation of the mammalian heart is influenced by knockout of either Tafazzin or Crls1 in mice. Tafazzin is a mitochondrial transacylase that is essential after initial cardiolipin (CL) biosynthesis for cardiolipin remodeling and the generation of highly unsaturated cardiolipin species. In contrast, Crls1, the cardiolipin synthase, is required for de novo cardiolipin biosynthesis.

The authors conclude that Crls1KO prevents the postnatal increase in mitochondrial cristae density and the accumulation of respiratory chain proteins, ultimately leading to death of mice within 15 days after birth. In contrast, tafazzin deletion did not impair mitochondrial maturation or postnatal cardiac development to a comparable extent. In a separate experiment, the authors show that the remodeling enzymes TAFAZZIN and ABHD18, but not other enzymes of the cardiolipin pathway, co-migrate in a complexome analysis with a nascent respiratory complex I intermediate, which is present during respiratory chain biogenesis.

Overall, this is a very well-executed experimental study with interesting findings. The main issue, in my view, is that the complexome analysis in Fig. 5 does not really fit with the rest of the story and instead represents the starting point for an independent project (see major points below). My suggestion would be to remove the complexome analysis from the manuscript and further develop it separately. In parallel, the core story regarding postnatal cardiac development could be strengthened by expanding the analyses of existing data and some additional experiments (see below). This would sharpen the scope of the study and represents an interesting contribution for the readership of EMBO reports.

Major points

1) One prominent issue is that the manuscript seems to have a kind of "identity crisis". While the first part deals with the role of cardiolipin biosynthesis and remodeling in postnatal cardiac development, the second complexome part that focusses on TAFAZZIN and ABHD18 feels like a completely different story and does not include Tafazzin/Crls1 knockout animals. While the first part is relatively descriptive but complete, the second, complexome-related part appears more like the beginning of a research project and is not sufficiently complete (see below).

2) Throughout all figures, the statistical method of choice has been unpaired t-tests without correction for multiple testing. However, most of the data can be considered time-series data, often involving more than two groups. The larger datasets (lipidomics, proteomics) are segmented into much smaller analyses, which does not necessarily eliminate the need for appropriate statistical consideration across all features and conditions. I would therefore strongly recommend consulting with a statistics expert and rework where needed.

3) To further strengthen the conclusions on the role of CL in mammalian heart maturation, it would be valuable to include analyses of female KO mice. As the manuscript primarily addresses enzymatic functionality rather than Barth Syndrome per se, inclusion of both sexes could broaden the relevance of the findings. Barth Syndrome being a X-linked disease is a weak argument for using male mice only.

4) In general, a more detailed description of the mouse cohorts is needed, including body weight progression over the 15-day period and food intake. This information is particularly important because Fig. 4C interprets changes in lipid droplet size and related parameters, which could reflect severe physiological stress in the Crls1KO mice resulting in less food intake rather than a specific Crls1 effect.

5) How do the authors explain the pronounced, although not statistically significant, differences in CL between Crls1Ctrl and TafazzinCtrl at days 1 and 3 (Fig. 1)?

6) The authors explain the "residual" CL content in the Crls1KO (which as far as I can see actually corresponds to near-normal levels) by arguing that the knockout occurs late during development. This explanation is not entirely convincing, as under the same experimental conditions the TafazzinKO already exhibits a pronounced phenotype as early as day 1 (Fig. 2D). Moreover, CL content in the Crls1KO remains constant over the full period of 15 days when normalized to protein content (Fig. 1B), while the total heart mass increases substantially during this time (Fig. 1E). Taken together, one would expect that residual CL would be diluted down over time, which it does not. Can the authors explain their observation by changes in mitochondrial volume or biomass, or are there alternative explanations? In this regard it would make sense to validate thier knockout efficiency by Western Blot and qPCR. This would also help to clarifying whether the remaining CL synthesis activity observed at days 3 and 13 indeed originates from residual Crls1.

7) The authors report surprisingly low tetralinoleoyl-CL levels, which then also decrease with age (Fig. 2C). This appears somewhat unexpected, as in adult mice tetralinoleoyl-CL is typically reported to be the dominant CL species in the heart. A discussion of possible explanations for this observation would be appreciated.

8) Lipid changes are interpreted quantitatively on a lipid class level (PC/PS, "less prominent", "more prominent", page 5). For making such claims (and I assume they are correct) the sum of all species of the respective class should be compared (as in Fig. 2B)

9) In Data S1, among the most strongly altered lipid species, a surprisingly high number of sphingomyelin species are present (especially in the TafazzinKO context), but they are not mentioned in the manuscript. It would be helpful to comment on this observation and clarify its relevance.

10) It would be informative to show MLCL and DLCL species in Fig. S2, particularly to illustrate changes in saturation/unsaturation. Also, in Data S1, a considerably larger number of CL species are presented, including MLCL, DLCL, and CL species >76:9. Please clarify the criteria used for selecting which species were plotted. In line with this, it is unclear whether the upper panel of Fig. S2 duplicates data shown in Fig. 2C. If so, duplication should be avoided or at least precisely explained.

11) On page 5 it is described that "Crls1KO decreases mitochondrial proteins". However, the respective Fig. 3A shows ratios between KO/WT which does not allow for such quantitative claims. The housekeeper normalized data in Fig. 3B shows that the behavior is more complex than that. Please describe more accurately.

12) Depending on cut-off criteria, the proteomic dataset either delivers a lot or almost no positive hits. The labelling of individual proteins in Fig. 3 seems to be quite selective. Why are some hits interpreted/labelled and others not? What were the exact criteria that guided the analysis of the proteomics data? Which cut-offs have been used? Was also a general analysis performed (for example gen set enrichments etc.) and why is this not shown in the supplement?

13) In the proteomics dataset, the main enzymes discussed in the manuscript (TAFAZZIN, CRLS1, and ABHD18) are not detected, whereas they are identified in the complexome analysis. A brief explanation for this discrepancy would be helpful.

14) Representative electron microscopy images corresponding to the analyses shown in Figure 4A should be included in the supplementary material to allow readers to better assess the original data from which the values have been derived from.

15) In Fig. 4B, Crls1Ctrl mice appear to have higher levels of all mitochondrial complexes, whereas Crls1 KO mice show levels comparable to both TafazzinCtrl and KO mice. This seems inconsistent with Fig. 3C, where TOM complex levels are similar but OXPHOS complexes are reduced. Further clarification or discussion of this apparent discrepancy would improve interpretability.

16) The authors do not show to what extent the assembly of respiratory supercomplexes is impaired in TafazzinKO and Crls1KO mice (e.g. day 3 vs. 13) and also do not assess respective functional consequences. An experiment addressing this point would nicely complement the previously described changes in lipid composition, proteomics, and morphology by providing functional insight.

17) The depiction of the complexome analysis is accompanied by very strong claims (e.g. "likely represents a partially assembled complex of the two enzymes"), that are not (yet) sufficiently supported by the presented data. The authors have not shown evidence for a direct association between individual proteins, but demonstrated co-migration in a gel. Direct binding or association has to be proven with additional orthogonal experiments. Consequently, the "In summary" paragraph at the end of page 7 is overinterpreted. While I agree with the authors that an association of ABHD18 with complex I may be plausible, a scientific manuscript requires experimental evidence rather than conjecture.

18) Traces for CRLS1, TAMM41 and PTPMT1 in the complexome analysis look more like noise rather than signals (Fig. 5F) and for PGS1 a substantial fraction of the signal is present in a region that is smaller than the protein size itself. The authors should clarify in detail what the quality criteria for their analysis of individual proteins were? For example, why was only ABHD18 included in the analysis, and not other accessory CL-cleaving enzymes that have been reported by the group in earlier work?

19) The method description of the complexome should be written in a transparent manner that allows full reproducibility.

20) In the discussion section, the statement that Crls1KO and TafazzinKO induce "similar degrees of CL deficiency" is not compatible with the data contained in the manuscript (CL pattern, MLCL accumulation, etc.). In a similar manner the later statement that "both strains were born with normal CL levels" is a very oversimplified view on the lipid data and might be misleading for the general reader.

21) Also in the discussion section, the claim that "TAFAZZIN and ABHD18 associate with the nascent respiratory chain" is not proven (see comments above).

Minor points:

- Throughout the manuscript Latin phrases and species names should be consistently written in italic.

- The depiction of CL remodeling in the introduction (page 2, last 3 lines) is so strongly simplified that it could, at best, be considered incomplete, or even misleading. Please describe it in a more precise and detailed manner. Also, a more comprehensive description of general cardiac development in mice would be useful for the reader of the manuscript.

- The y-axis in Fig. 1A needs a proper label (percentage) that ensures it is not confused with the number of mice (N =). Since the number of mice is provided above each bar, clarifying or relabeling the axis would improve readability.

- Use the same scale bar size for all depictions of day 13 in Fig. 1E.

- In Fig 2: A requires a legend for all lipid classes. The arrangement of D as it is at the moment is a bit confusing/nonlogical. The figure legend is not informative enough. The label in C is moved upwards in a way that it is not clear if it belongs to the panel or not.

- In Data S1 Crls1Ctrl and KO a single MLCL (CL(14:1_24:1_24:1) is not included in the MLCL sum. Can the authors comment on why this is the case?

- On page 6: "estimated concentrations of specific proteins". Please clarify that this is done on the basis of the same proteomics data as before. Update the methods section with an exact description of how you have done that (reproducible) instead of the one-liner.

- In Fig. 3A at 4-8 days a red dot is present that might correspond to "Mitochondrial". This should be added to the upper legend as well.

- The arrangement of panels in Fig 4 is non-logical (A-C-B). Some formatting errors in axis labels.

- Fig. 4A it not clear which time points are included in 0-5, 5-10, 10-15 and should be changed to e.g. 0-4,5-9,10-15.

- Please describe in detail which mice have been used for the complexome analysis (in respect to the control mice in the previous parts, compare also respective comment in major points).

Referee #1:

Ren et al. present a very interesting study showing that cardiomyocyte-restricted deletion of Crls1 (CL synthase) but not Tafazzin (remodeling) disrupts postnatal cardiac maturation, preventing the normal rise in cristae density and intra-mitochondrial concentration of respiratory proteins. Consequently, animals develop heart failure and died by ~P16. Conversely, Tafazzin loss reduces CL remodeling but does not block early postnatal maturation. The authors propose a mechanistic explanation: TAFAZZIN and ABHD18 (a CL lipase) co-migrate with nascent complex I assemblies (assessed by complexome profiling of P13 neonatal heart mitochondria) whereas de novo biosynthetic enzymes (PGS1/TAMM41/PTPMT1/CRLS1) do not-suggesting remodeling enzymes transiently associate with complex I precursors.
Those conclusions are based on lipidomic and proteomic analysis together with cardiac function studies and electron microscopy analysis of the cardiac mitochondria. The lipidomic studies performed in 2-day intervals across the first 2 postnatal weeks showed that:
CL rises in controls but not in either KO.
MLCL/DLCL accumulate in Tafazzin_KO, whereas PG accumulates in Crls1_KO.
CL species become longer/more unsaturated with age in WT; this maturation is blocked or slowed in the KOs.
The proteomics analysis shows:
A strong age-dependent increase of OXPHOS complexes, while TOM complex remains stable, in WT. This is abrogated in Crls1_KO (but not in Tafazzin_KO)
ASNS increases in both KOs.
The Cardiac function study found:
Dilated cardiomyopathy in Crls1_KO, but not in Tafazzin_KO during neonatal period.
The EM analysis revealed an increase in cristae density during weeks 1-2 in WT and Tafazzin_KO but not in Crls1_KO. It also showed a sharp drop in lipid droplets/TG in Crls1_KO.
The work is concise yet data-rich, with clear physiological relevance and a mechanistic angle on membrane biogenesis/respiratory chain assembly. The side-by-side comparison of de novo synthesis vs remodeling in postnatal heart maturation is timely and conceptually novel. The finding that CL synthesis (CRLS1)-but not remodeling-controls the age-dependent rise of respiratory protein concentration and cristae expansion in vivo is mechanistically intriguing and of broad interest to mitochondrial biology and developmental physiology. However, it may need targeted additional evidence and improvements in statistical reporting and figure clarity to be fully convincing.
RESPONSE: We appreciate the feedback and the detailed criticism. In particular, we are very grateful for suggestions to improve the statistical treatment of the data.

Major points:
1. Statistics and multiple testing across omics are required: The Volcano plots use p<0.01 with a 4-fold cutoff but there's no multiple-testing correction stated for hundreds to thousands of features. Please report adjusted q-values for all lipid and protein differential analyses; ensure effect sizes and confidence intervals accompany bar/line plots. With respect to the age × genotype designs sampled longitudinally (2-day intervals), a linear mixed-effects model (or at minimum two-way ANOVA with interaction and proper post-hoc FDR correction) is more appropriate than multiple unpaired t-tests. Clarify the exact model and assumptions (normality/variance).

RESPONSE: Statistical analysis was revised extensively throughout the manuscript. We carried out mixed-effects analysis by the restricted maximum likelihood method for age-dependent data in Figs 1-4, applying a full model with interactive effects and Geisser-Greenhouse correction (default parameters of the GraphPad Prism 10). Genotypes were compared at each age by the Bonferroni method. Significance threshold of adjusted P-values was set to 0.05. In volcano plots (Figs 2A, 3A), appropriate significance levels were determined by the Benjamini-Hochberg procedure using a false discovery rate of 0.05. The significance threshold was $10^{-1.99}$ for lipidomics data and $10^{-2.17}$ for proteomics data. Consequently, P<0.01 was considered significant in lipidomics volcano plots and P<0.001 was considered significant in proteomics volcano plots. A "Statistical analysis" section was added to Methods.

2. The statement: "associate with nascent complex I" is not fully supported by the data that only support that "co-migrate with complex I assembly intermediates". This shouls be correct throughout the text unless additional evidence is provided The co-migration of TAFAZZIN/ABHD18 with complex I precursors is suggestive but not yet definitive evidence for physical interaction or functional coupling; this is correlative (complexome profiling). The language should be tempered or supported by orthogonal approaches. Complexome profiling shows co-migration, not binding. To consolidate this claim additional independent experimental confirmation is needed. Examples of those approaches may be Proximity labeling (APEX2/BioID) of complex I assembly intermediates to capture TAZ/ABHD18 enrichment. Crosslinking-MS or co-IP from digitonin-solubilized mitochondria under conditions that preserve the assembly intermediates. Replicate complexomes of the Tafazzin_KO and or ABHD18_KO showing that the mobility and/or the amount of the proposed CI interacting partners is affected when compared with control and Crls1_KO complexomes.

RESPONSE: We agree. Following the editor's suggestion, the complexome profiling data were removed from the manuscript.

3. Survival and echocardiography presentation
Figure 1A: Replace genotype frequency vs age with a Kaplan-Meier survival curve (include N per group, events, censoring, log-rank p-value, and hazard ratio with 95% CI). The text states ~16-day mean lifespan for Crls1_KO-this belongs in a formal survival analysis.
Figure 1F (echo): Show representative M-mode images and provide full echo parameters (LVEDD, LVESD, LVFS, EF if measured, heart rate under anesthesia, temperature), plus n and exact p-values. The methods are solid and blinded, but readers must see the data.

RESPONSE: Data for Fig 1A were not collected by longitudinal cohort observation as required for Kaplan-Meier analysis. Instead, we genotyped mouse litters at different ages and calculated the proportion of respective genotypes. From such data, the probability of a genotype being alive at a certain age can be calculated. If gene deletion has no effect on survival (null hypothesis), 50% of homozygous littermates are expected to be knockouts. We revised Fig 1A to harmonize the presentation of the 2 KO models and applied binomial testing, which determines the probability of type 1 error in the comparison of proportions. M-mode images were not collected. Instead, all measurements were made from 2-D images (Phoon et al, Mouse Cardiovascular Imaging, Current Protocols, 4, e1116. doi: 10.1002/cpz1.1116, 2024). LVEDD and LVFS are shown in Figure 1F. LVESD did not show consistent trends. EF is typically not measured but can be calculated from the shortening fraction using formulas that have certain geometric assumptions. We prefer to present shortening fractions rather than quantities derived from them (Fig 1G).

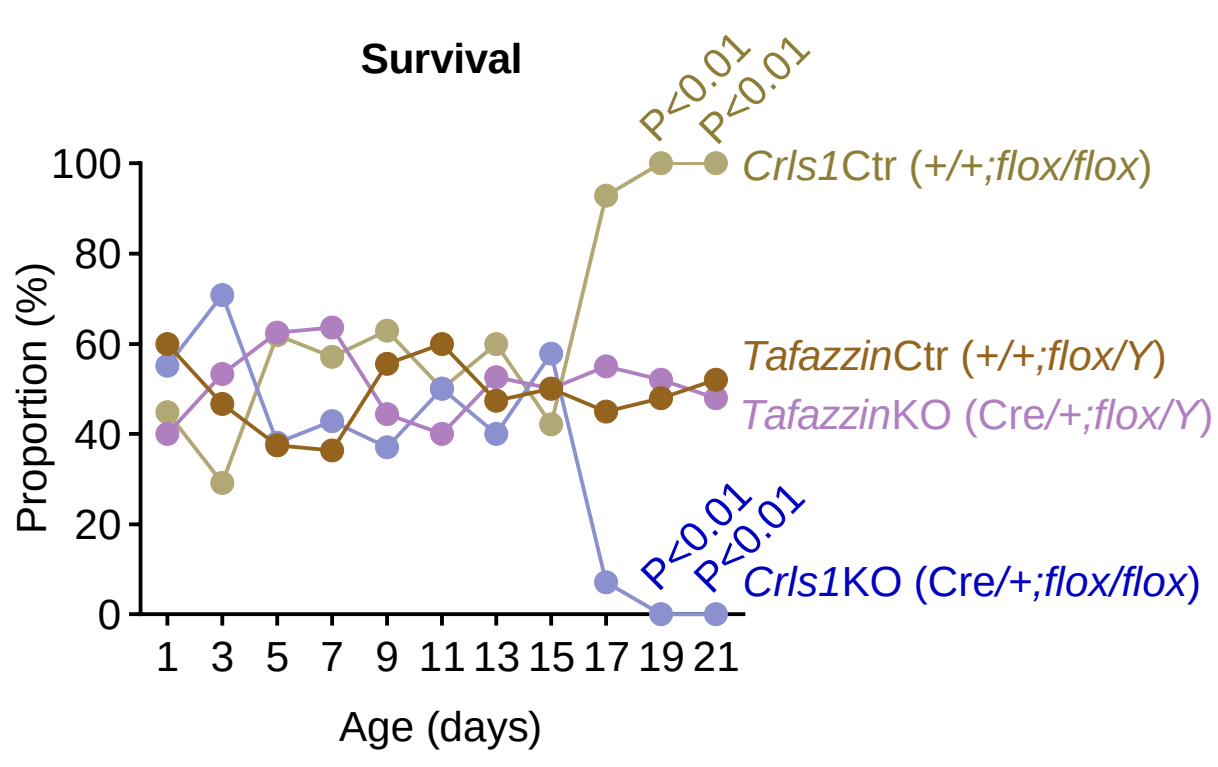

Fig 1A

4. Sex as a biological variable. The authors state analyses were done only in males because Tafazzin is X-linked, yet Figure 1A labels "Crls1 (male & female)." Please clarify and harmonize the sex used per genotype across all experiments. Thus provide a brief analysis showing no sex effect in Crls1_KO or restrict to males consistently.

RESPONSE: Female data were removed from the graph. The two mouse models were combined in a single graph presenting only KO and control (Fig 1A). We established that sex did not affect survival in Crls1KO mice (P=0.7031, analysis of variance) and stated this fact in the Results section of the revised manuscript.

5. Clarify sampling and material used for each assay. Specify, for each figure, whether lipids/proteins were measured in whole heart homogenate, purified mitochondria, or isolated cardiomyocytes (only CL at P3 cardiomyocytes is explicitly stated). The lipidomics/proteomics/EM sections should clearly map to tissue fraction and age.

RESPONSE: Changes were made as suggested. Study material is specified in subheadings in Fig 1 and in the legends of Figs 2-4. The methods sections were also amended.

6. Causal link between CRLS1 loss and suppressed OXPHOS within mitochondria. The authors infer reduced intra-mitochondrial concentrations of OXPHOS from LFQ/TOM normalization. Consider corroborating with: BNPAGE quantification of complexes and supercomplexes; enzymatic activities (CI-CV) and oxygen consumption in permeabilized fibers or isolated cardiomyocytes; or mitochondrial mass markers (mtDNA copy number, citrate synthase) to disentangle content vs concentration changes.

RESPONSE: We agree but unfortunately, additional samples are not available to allow these confirmatory measurements across genotypes and ages.

7. Interpreting lipidomic differences mechanistically need to be tempered. The PG accumulation (Crls1_KO) vs MLCL/DLCL accumulation (Tafazzin_KO) is compelling. But the solid mechanistic interpretation may require to be strengthened by testing whether PG elevation per se can depress OXPHOS expression/assembly (e.g., PG supplementation in cardiomyocytes or genetic PGS1 perturbation), or by rescue with CRLS1 re-expression in Crls1_KO background (even partial, AAV if feasible). I am not thinking that these experiments are required for this manuscript, but the claim should be tempered.

RESPONSE: In the revised manuscript, these explanations are explicitly marked as speculations (3rd paragraph of the Discussion).

8. CRLS1 activity assay validation. The assay uses PG14:0/14:0 + CDPDG18:1/18:1 to form CL14:0/14:0/18:1/18:1, a non-endogenous product-as you note in Figure S1. It

would be convenient to add a standard curve and LOD/LOQ and specificity controls (e.g., heat-inactivation; metal chelation).

RESPONSE: Standard curves are now included in Figure EV1 of the revised manuscript. Blanks were created by adding organic solvents before the initiation of the reaction. The blank signal was 0.549±0.266 pmol CL14:0/14:0/18:1/18:1 (N=4), which makes the limit of blank 0.987 pmol (mean+1.645*SD). We amended the legend to reflect this.

9. Figures need to be improved for clarity and better understanding.
Figure 1 (phenotypes & survival/echo) required legend refinement: Explicitly define what each panel represents. State n per time point, statistical test, two-tailed, and report exact p. Add Kaplan-Meier as noted; include representative M-mode frames and scale bars on histology.
Figure 2 (lipidomics): For volcano plots, display FDR-adjusted significance ($q<0.05$) and label key features (PG, CL, MLCL, DLCL). Add a methods inset clarifying sample source (whole heart vs mitochondria). In species plots and unsaturation/chain-length trajectories, include n per age, CIs, and exact p-values.
Figure 3 (proteomics): Clearly indicate how "OXPHOS/TOM" ratios were computed (sum of LFQs for subunits listed in Data S2?). Consider adding a heatmap of complex I/III/IV/V subunits over age for each genotype (z-scored per protein), with FDR. Include rationale for MYH6/MYH7 ratio as maturation marker and consider additional maturation markers more related with OXPHOS like the expression of paralogue genes for Complex IV related with cardiomyocyte maturation.
Figure 4 (EM and droplets/TG): Show representative EM images for each genotype and age (multiple magnifications), with quantification overlay. Provide blinding statement for EM counting. Ensure lipid droplet density is measured across comparable tissue regions.
Figure 5 (complexome): Provide replicate overlays of TAZ/ABHD18 peaks with ECSIT/TMEM126B/TMEM186 to visualize co-migration; add mass ladder ticks to the x-axis across all panels for ease of interpretation. Clarify digitonin ratio (6 g/g) rationale and whether alternate detergents were tested.
Supplementary Figures S1-S2
S1 (CRLS1 assay): add time course and protein titration plots with linear fits and r^2, plus the controls suggested above.
S2 (age-dependent CL species): enrich with stacked barplots or ridge plots across ages; indicate N=3 in the panel and include exact species lists in Data S1 for reproducibility.
RESPONSE: The following revisions were made:

- Study material and statistics were defined in the legend of Fig 1 & 2

- An explanation of how OXPHOS/TOM ratio was calculated was provided in the Results section
- EM images were added to Fig 4
- A blinding statement was added to the Methods section
- Fig 5 was deleted
- Linear regression analysis was included in Fig EV1
- N=3 was specified in the legend of Fig EV2 but for the sake of consistency, the molecular group notation was maintained in Fig EV2 (molecular groups may contain more than one molecular species and those species may be different across genotypes for the same molecular group)

Maturation markers other than MYH6 and MYH7 were not consistently identified in the proteomics data to allow their application in this study.

Referee #2:

In the manuscript by Ren et al. the authors describe how cardiolipin is affecting cardiomyocyte maturation in the mammalian heart. It is a novel study that is well described and the authors made good graphical illustrations of their findings. I only have minor but important comments to address in the manuscript:

RESPONSE: We appreciate the comments and revised the manuscript accordingly.

In figure 1, the authors show the mouse hearts in the different genetypes. It seems that the KO mice have smaller hearts than the control groups. Was the heart tissue weighed? And if yes, was there any differences?

RESPONSE: Weights of mouse hearts are now presented in Fig 1H of the revised manuscript. Weights of *Crls1*KO hearts were higher than their controls on day 13 but not on day 3. There was no difference between *Tafazzin*KOs and controls.

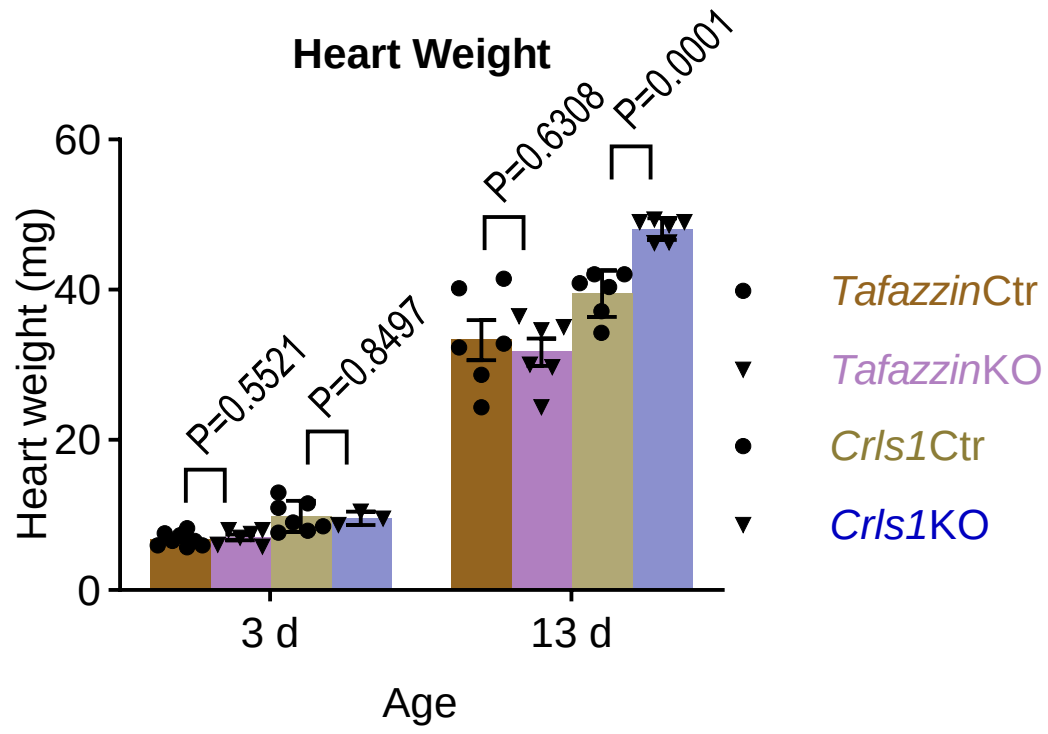

Fig 1H

It is not fully clear how many mice there were in each group in the analyses performed. In figure 2 legend, it is mentioned that 6 biological replicas per genotype and age group were used, however for some of the omics performed i.e. figure 3 B-E only N=3, but also for the lipidomics etc? Why was not all 6 mice used for the omics to strengthen the statistics?

RESPONSE: We analyzed 3 biological replicas per time point. For each volcano plot, data from 2 time points (6 replicas) were pooled. This is now clarified in the legends of Figs 2 & 3.

Another comment to the data analysis is how well represented the respiratory chain proteins are represented in the proteomics. This should be clear in the legends of the figures i.e. figure 3, 4 and 5.

RESPONSE: All subunits included in the analysis of the respiratory chain are listed in the proteomics data set (Table EV2). This is now explicitly stated in the legend of Fig 3.

For the discussion, it would be relevant to discuss the differences between human and mouse cardiomyocyte maturation and compare this to their own findings. Also, the findings that Tafazzin variants does not lead to affect cardiac function until the mice reaches adulthood, whereas the human disease often manifests during early childhood? The authors should discuss this in the manuscript.

RESPONSE: The following paragraph was added to the discussion: "Differences exist between mouse and human cardiomyocytes, most notably in the timing of the proliferation-to-hypertrophy transition and in the number of nuclei per cell (Guo & Pu, 2020), which may limit the human relevance of our data. Patients with *Tafazzin* mutations (Barth syndrome) often develop cardiomyopathy in infancy (Kang *et al*, 2016; Roberts *et al*, 2012) whereas TAFAZZIN-deficient mice develop cardiomyopathy only after weaning regardless of whether *Tafazzin* is deleted in the whole body or only in cardiomyocytes (Wang *et al*, 2020; Zhu *et al*, 2021)."

Referee #3:

Ren et al. investigated how postnatal maturation of the mammalian heart is influenced by knockout of either Tafazzin or Crls1 in mice. Tafazzin is a mitochondrial transacylase that is essential after initial cardiolipin (CL) biosynthesis for cardiolipin remodeling and the generation of highly unsaturated cardiolipin species. In contrast, Crls1, the cardiolipin synthase, is required for de novo cardiolipin biosynthesis.

The authors conclude that Crls1KO prevents the postnatal increase in mitochondrial cristae density and the accumulation of respiratory chain proteins, ultimately leading to death of mice within 15 days after birth. In contrast, tafazzin deletion did not impair mitochondrial maturation or postnatal cardiac development to a comparable extent. In a separate experiment, the authors show that the remodeling enzymes TAFAZZIN and ABHD18, but not other enzymes of the cardiolipin pathway, co-migrate in a complexome analysis with a nascent respiratory complex I intermediate, which is present during respiratory chain biogenesis.

Overall, this is a very well-executed experimental study with interesting findings. The main issue, in my view, is that the complexome analysis in Fig. 5 does not really fit with the rest of the story and instead represents the starting point for an independent project (see major points below). My suggestion would be to remove the complexome analysis from the manuscript and further develop it separately. In parallel, the core story regarding postnatal cardiac development could be strengthened by expanding the analyses of existing data and some additional experiments (see below). This would sharpen the scope of the study and represents an interesting contribution for the readership of EMBO reports.

RESPONSE: We are very grateful for the thoughtful comments. The manuscript was revised extensively. Complexome analysis was removed from the manuscript as suggested.

Major points

1) One prominent issue is that the manuscript seems to have a kind of "identity crisis". While the first part deals with the role of cardiolipin biosynthesis and remodeling in postnatal cardiac development, the second complexome part that focusses on TAFAZZIN and ABHD18 feels like a completely different story and does not include Tafazzin/Crls1 knockout animals. While the first part is relatively descriptive but complete, the second, complexome-related part appears more like the beginning of a research project and is not sufficiently complete (see below).

RESPONSE: We agree. The complexome profiling data were removed.

2) Throughout all figures, the statistical method of choice has been unpaired t-tests without correction for multiple testing. However, most of the data can be considered time-series data, often involving more than two groups. The larger datasets (lipidomics, proteomics) are segmented into much smaller analyses, which does not necessarily eliminate the need for appropriate statistical consideration across all features and conditions. I would therefore strongly recommend consulting with a statistics expert and rework where needed.

RESPONSE: Statistics was revised extensively, as described in a new paragraph of the revised manuscript ("Statistical analysis" under Methods): "Data are biological replicas collected at multiple neonatal ages (1-15 days). Samples were analyzed in two separate runs, one for the *Tafazzin*Ctrl and *Tafazzin*KO pair and another for the *Crls1*Ctr and *Crls1*KO pair. Data were analyzed by the software GraphPad Prism 10. Comparisons between groups were made by t-test (unpaired, two-tailed). The proportion of genotypes at different ages was analyzed by binomial test (two-tailed). To evaluate age-dependent progression of variables in different genotypes, mixed-effects analysis was performed by the restricted maximum likelihood method, applying a full model with interactive effects and Geisser-Greenhouse correction (default parameters of the GraphPad Prism 10). Genotypes were compared at each age by the Bonferroni method. Significance threshold of adjusted P-values was set to 0.05. In volcano plots, the significance level was determined by the Benjamini-Hochberg procedure using a false discovery rate of 0.05. The significance threshold was $10^{-1.99}$ for lipidomics data and $10^{-2.17}$ for proteomics data. Consequently, $P<0.01$ was considered significant in lipidomics volcano plots and $P<0.001$ was considered significant in proteomics volcano plots."

3) To further strengthen the conclusions on the role of CL in mammalian heart maturation, it would be valuable to include analyses of female KO mice. As the manuscript primarily addresses enzymatic functionality rather than Barth Syndrome per se, inclusion of both sexes could broaden the relevance of the findings. Barth Syndrome being a X-linked disease is a weak argument for using male mice only.

RESPONSE: We agree that conclusions about the role of cardiolipin in cardiomyocyte maturation would be strengthened by the inclusion of female mice. However, samples from female mice were not collected because tafazzin deletion only affects males. It was not the goal of this study to determine the effect of sex. Such endeavor, would require at least 2 but probably more like 3-4 times the sample size of the present study, which already stands at 96 mice.

4) In general, a more detailed description of the mouse cohorts is needed, including

body weight progression over the 15-day period and food intake. This information is particularly important because Fig. 4C interprets changes in lipid droplet size and related parameters, which could reflect severe physiological stress in the Crls1KO mice resulting in less food intake rather than a specific Crls1 effect.

RESPONSE: It is difficult to determine food intake in mouse pups before weaning as they rely completely on breast feeding. Therefore, food intake was not measured. However, body weights (Fig 1B) and heart weights (Fig 1H) were added to the revised manuscript.

5) How do the authors explain the pronounced, although not statistically significant, differences in CL between Crls1Ctrl and TafazzinCtrl at days 1 and 3 (Fig. 1)?

RESPONSE: We can only speculate but would like to point out that lipid analyses were run in 2 batches, each containing a KO and its control. Consequently, the two controls were analyzed many months apart, which could be the reason for the discrepancy. In any case, the difference between CL concentrations in *Crls1*Ctr and *Tafazzin*Ctr was not statistically significant (P=0.8775 on day 1 and P=0.4274 on day 3, after Bonferroni adjustment of P-values in mixed-effects analysis).

6) The authors explain the "residual" CL content in the Crls1KO (which as far as I can see actually corresponds to near-normal levels) by arguing that the knockout occurs late during development. This explanation is not entirely convincing, as under the same experimental conditions the TafazzinKO already exhibits a pronounced phenotype as early as day 1 (Fig. 2D). Moreover, CL content in the Crls1KO remains constant over the full period of 15 days when normalized to protein content (Fig. 1B), while the total heart mass increases substantially during this time (Fig. 1E). Taken together, one would expect that residual CL would be diluted down over time, which it does not. Can the authors explain their observation by changes in mitochondrial volume or biomass, or are there alternative explanations? In this regard it would make sense to validate thier knockout efficiency by Western Blot and qPCR. This would also help to clarifying whether the remaining CL synthesis activity observed at days 3 and 13 indeed originates from residual Crls1.

RESPONSE: We agree that he knockout efficiency for TAFAZZIN appeared to be greater than the knockout efficiency for CRLS1 as assessed by CL composition (Fig 2D). In the revised discussion, "…we speculate that different half-lives of TAFAZZIN and CRLS1 are responsible for this discrepancy. TAFAZZIN has a very short half-life in mammalian cell cultures (Xu *et al*, 2015), whereas most other proteins of the inner mitochondrial membrane are known for their longevity (Bomba-Warczak *et al*, 2021; Krishna *et al*, 2021)." Western blots would indeed be helpful to verify Knockout efficiencies but suitable antibodies are not available. Instead, we verified knockout

efficiency by measuring enzymatic activity, which in our mind is most relevant as it determines function rather than protein concentration or message. These measurements demonstrate that CRLS1 activity was reduced by 50-60% at birth and then declined further (Fig 1E). The remaining CRLS1 activity was obviously sufficient to maintain a constant CL concentration in a growing heart, which, as the reviewer noticed, requires net accumulation of CL per heart. We do not see any contradiction because even low enzymatic activities may sustain a certain fraction of the normal rate. Regardless, the net production of CL in the Crls1KO remained way below normal (Fig 1C). In the revised manuscript, we no longer use the term “residual CL” because, as the reviewer noted, it detracts from the fact that there is ongoing CL synthesis.

7) The authors report surprisingly low tetralinoleoyl-CL levels, which then also decrease with age (Fig. 2C). This appears somewhat unexpected, as in adult mice tetralinoleoyl-CL is typically reported to be the dominant CL species in the heart. A discussion of possible explanations for this observation would be appreciated.

RESPONSE: A short comment was included to draw attention to the decline in tetralinoleoyl-CL (CL72:8) during maturation. We are unsure about the explanation but would like to point out that there is considerable variability in the CL composition caused by different mouse strains and feeding conditions (Int J Mol Sci. 2012 Nov 21;13(11):15447–15463, Biochemistry 2007 May 29;46(21):6417-6428). Also, little is known about the cardiolipin trajectories during postnatal maturation, which could be an intriguing avenue for future research.

8) Lipid changes are interpreted quantitatively on a lipid class level (PC/PS, "less prominent", "more prominent", page 5). For making such claims (and I assume they are correct) the sum of all species of the respective class should be compared (as in Fig. 2B)

RESPONSE: We agree and re-worded: “With increasing age, more PC and PS species changed their concentration. The total PI concentration was abnormally low at birth but increased back to normal by postnatal day 9 (Figure 2B).”

9) In Data S1, among the most strongly altered lipid species, a surprisingly high number of sphingomyelin species are present (especially in the TafazzinKO context), but they are not mentioned in the manuscript. It would be helpful to comment on this observation and clarify its relevance.

RESPONSE: We mention sphingomyelin in the revised manuscript but would like to stay away from speculations about its relevance.

10) It would be informative to show MLCL and DLCL species in Fig. S2, particularly to

illustrate changes in saturation/unsaturation. Also, in Data S1, a considerably larger number of CL species are presented, including MLCL, DLCL, and CL species >76:9. Please clarify the criteria used for selecting which species were plotted. In line with this, it is unclear whether the upper panel of Fig. S2 duplicates data shown in Fig. 2C. If so, duplication should be avoided or at least precisely explained.

RESPONSE: The duplication was removed from Fig EV2. Compositions of MLCL, DLCL (for TafazzinKO), and phosphatidylglycerol (for Crls1KO) were added to Fig EV2. Only major molecular groups (abundances>2%) were included in the graphs. This is now explicitly stated in the legend of Fig 2C and Fig EV2 of the revised manuscript.

11) On page 5 it is described that "Crls1KO decreases mitochondrial proteins". However, the respective Fig. 3A shows ratios between KO/WT which does not allow for such quantitative claims. The housekeeper normalized data in Fig. 3B shows that the behavior is more complex than that. Please describe more accurately.

RESPONSE: The sentence was changed to: "Most prominently, *Crls1*KO decreased the abundance of several mitochondrial proteins and increased the abundance of several enzymes of the amino acid metabolism in an age-dependent manner (Figure 3A)."

12) Depending on cut-off criteria, the proteomic dataset either delivers a lot or almost no positive hits. The labelling of individual proteins in Fig. 3 seems to be quite selective. Why are some hits interpreted/labelled and others not? What were the exact criteria that guided the analysis of the proteomics data? Which cut-offs have been used? Was also a general analysis performed (for example gen set enrichments etc.) and why is this not shown in the supplement?

RESPONSE: In the revised manuscript, the appropriate significance level was determined by the Benjamini-Hochberg procedure using a false discovery rate of 0.05. The significance threshold was $10^{-1.99}$ for lipidomics data and $10^{-2.17}$ for proteomics data. Consequently, P<0.01 was considered significant in lipidomics volcano plots and P<0.001 was considered significant in proteomics volcano plots. Lipids and proteins that meet these criteria are labeled if they belong to the specified category.

13) In the proteomics dataset, the main enzymes discussed in the manuscript (TAFAZZIN, CRLS1, and ABHD18) are not detected, whereas they are identified in the complexome analysis. A brief explanation for this discrepancy would be helpful.

RESPONSE: Complexome analysis was performed in purified mitochondria, which yields deeper coverage of mitochondrial proteins than can be achieved in total heart tissue. However, the complexome data were removed from the manuscript.

14) Representative electron microscopy images corresponding to the analyses shown in Figure 4A should be included in the supplementary material to allow readers to better assess the original data from which the values have been derived from.

RESPONSE: EM images are now included in the revised Fig 4.

15) In Fig. 4B, Crls1Ctrl mice appear to have higher levels of all mitochondrial complexes, whereas Crls1 KO mice show levels comparable to both TafazzinCtrl and KO mice. This seems inconsistent with Fig. 3C, where TOM complex levels are similar but OXPHOS complexes are reduced. Further clarification or discussion of this apparent discrepancy would improve interpretability.

RESPONSE: Because of the large number of samples, proteomics analysis was performed in two batches, one with all samples of the *Tafazzin*Ctrl/*Tafazzin*KO mice and the other with all samples of the *Crls1*Ctr/*Crls1*KO mice. In general, the ratios tended to be higher in the *Crls1*Ctr/*Crls1*KO batch than in the *Tafazzin*Ctrl/*Tafazzin*KO batch. Comparison between batches can produce false discoveries. Therefore, we did not draw any conclusions from the discrepancy but revised the Methods section to specify that samples were analyzed in two separate batches.

16) The authors do not show to what extent the assembly of respiratory supercomplexes is impaired in TafazzinKO and Crls1KO mice (e.g. day 3 vs. 13) and also do not assess respective functional consequences. An experiment addressing this point would nicely complement the previously described changes in lipid composition, proteomics, and morphology by providing functional insight.

RESPONSE: Additional samples are not available to carry out such experiments. Also, the effect of CL on respiratory supercomplexes has been well documented by us and others (for instance: J Mol Biol 361:462-469, 2006; Nature Chem Biol 12:641-647, 2016).

17) The depiction of the complexome analysis is accompanied by very strong claims (e.g. "likely represents a partially assembled complex of the two enzymes"), that are not (yet) sufficiently supported by the presented data. The authors have not shown evidence for a direct association between individual proteins, but demonstrated co-migration in a gel. Direct binding or association has to be proven with additional orthogonal experiments. Consequently, the "In summary" paragraph at the end of page 7 is overinterpreted. While I agree with the authors that an association of ABHD18 with complex I may be plausible, a scientific manuscript requires experimental evidence rather than conjecture.

RESPONSE: We agree. Complexome profiling data were removed. We will continue to study protein complex association of TAFAZZIN and ABHD18.

18) Traces for CRLS1, TAMM41 and PTPMT1 in the complexome analysis look more like noise rather than signals (Fig. 5F) and for PGS1 a substantial fraction of the signal is present in a region that is smaller than the protein size itself. The authors should clarify in detail what the quality criteria for their analysis of individual proteins were? For example, why was only ABHD18 included in the analysis, and not other accessory CL-cleaving enzymes that have been reported by the group in earlier work?

RESPONSE: See above. Complexome profiling data were removed.

19) The method description of the complexome should be written in a transparent manner that allows full reproducibility.

RESPONSE: Complexome analysis was deleted.

20) In the discussion section, the statement that Crls1KO and TafazzinKO induce "similar degrees of CL deficiency" is not compatible with the data contained in the manuscript (CL pattern, MLCL accumulation, etc.). In a similar manner the later statement that "both strains were born with normal CL levels" is a very oversimplified view on the lipid data and might be misleading for the general reader.

RESPONSE: Both sentences were deleted from the revised Discussion.

21) Also in the discussion section, the claim that "TAFAZZIN and ABHD18 associate with the nascent respiratory chain" is not proven (see comments above).

RESPONSE: This part was deleted as well.

Minor points:

- Throughout the manuscript Latin phrases and species names should be consistently written in italic.

RESPONSE: Latin phrases are written in Italic now.

- The depiction of CL remodeling in the introduction (page 2, last 3 lines) is so strongly simplified that it could, at best, be considered incomplete, or even misleading. Please describe it in a more precise and detailed manner. Also, a more comprehensive

description of general cardiac development in mice would be useful for the reader of the manuscript.

RESPONSE: The Introduction was revised accordingly.

First paragraph: "Mammalian heart development can be divided into 3 phases. The first phase, called specification, differentiates mesodermal progenitors into cardiac-specific lineages. The second phase, called morphogenesis, creates and connects the structural components of the heart. The third and final phase, called maturation, conditions the heart for lifelong contractions to sustain uninterrupted circulation. Maturation begins around birth and continues into the neonatal period. It alters cardiomyocytes through a tightly scripted program affecting all cellular compartments. Mitochondria, in particular, undergo profound changes in morphology and function but the mechanisms that control mitochondrial maturation are incompletely understood (Dimasi *et al*, 2023; Guo & Pu, 2020; Sakamoto & Kelly, 2024)."

Third paragraph: "CL is synthesized from phosphatidic acid at the inner leaflet of the inner membrane of mitochondria (Schlame & Haldar, 1993). The pathway requires 4 enzymes including phosphatidate cytidylyltransferase (TAMM41), CDP-diacylglycerol:glycerol-3-phosphate 3-phosphatidyltransferase (PGS1), phosphatidylglycerophosphatase (PTPMT1), and cardiolipin synthase (CRLS1) (Ren *et al*, 2014). *De novo* synthesis is followed by fatty acid remodeling, which exchanges the acyl chains for unsaturated fatty acids. Remodeling is initiated by the lipase ABHD18 that removes fatty acids from CL (Ren *et al* 2025; Masud *et al*, 2025). New and mostly unsaturated fatty acids are then transferred from other phospholipids to monolyso-cardiolipin (MLCL) by the acyltransferase TAFAZZIN (Xu *et al* 2006). This step regenerates CL and changes its composition of molecular species."

- The y-axis in Fig. 1A needs a proper label (percentage) that ensures it is not confused with the number of mice (N =). Since the number of mice is provided above each bar, clarifying or relabeling the axis would improve readability.

RESPONSE: Label was added to the y-axis of Fig 1A.

- Use the same scale bar size for all depictions of day 13 in Fig. 1E.

RESPONSE: Images in Fig 1F were re-scaled to a single scale bar.

- In Fig 2: A requires a legend for all lipid classes. The arrangement of D as it is at the moment is a bit confusing/nonlogical. The figure legend is not informative enough. The label in C is moved upwards in a way that it is not clear if it belongs to the panel or not.

RESPONSE: A legend specifying the color coding of lipid classes was added to Fig 2A. Panels were re-arranged. The legend was revised to include more procedural and statistical information.

- In Data S1 Crls1Ctrl and KO a single MLCL (CL(14:1_24:1_24:1) is not included in the MLCL sum. Can the authors comment on why this is the case?

RESPONSE: This species was not included because it is likely a minor CL species with incomplete assignment of MS2 fragments.

- On page 6: "estimated concentrations of specific proteins". Please clarify that this is done on the basis of the same proteomics data as before. Update the methods section with an exact description of how you have done that (reproducible) instead of the one-liner.

RESPONSE: The method is described in more detail in the Results section: "Next, we estimated the intramitochondrial concentration of respiratory complexes, ribosomes, and matrix enzymes. To this end we added the label-free quantitation (LFQ) intensities of their subunits (specified in Table EV2) and divided the sum by the LFQ intensity of the TOM complex (sum of the TOM subunits, Table EV2). Since the abundance of TOM is an indicator of the total surface area of mitochondria, the ratio becomes a measure of intramitochondrial concentration."

- In Fig. 3A at 4-8 days a red dot is present that might correspond to "Mitochondrial". This should be added to the upper legend as well.

RESPONSE: This issue became obsolete after revisions.

- The arrangement of panels in Fig 4 is non-logical (A-C-B). Some formatting errors in axis labels.

RESPONSE: Panels of Fig 4 were re-arranged.

- Fig. 4A it not clear which time points are included in 0-5, 5-10, 10-15 and should be changed to e.g. 0-4,5-9,10-15.

RESPONSE: The labels were changed (0-5, 6-9, 10-15).

- Please describe in detail which mice have been used for the complexome analysis (in respect to the control mice in the previous parts, compare also respective comment in major points).

RESPONSE: Complexome analysis was deleted.

Dear Dr. Schlame

Thank you for the submission of your revised manuscript to EMBO reports. I sincerely apologize for the unusual delay in the review process. Unfortunately, former referee #3 was unresponsive, despite having raised a number of important concerns. I have therefore asked referee #2 to evaluate the revised manuscript taking referee #3's concerns into account as well. We have now received the full set of referee reports that is copied below.

As you will see, both referees are very positive about the study and request only minor changes to clarify text and figures.

From the editorial side, there are also a few things that we need before we can proceed with the official acceptance of your study.

- Your study will be published in our Reports section. To make this possible, I kindly ask you to combine the Results and Discussion section.

- The Data availability paragraph should be placed before the Acknowledgments.

- Please provide the specific URLs for MSV000099296, PXD068888 datasets in the data availability statement.

- Please update the 'Conflict of interest' paragraph to our new 'Disclosure and competing interests statement'. For more information see
https://link.springer.com/journal/44319/submission-guidelines#conflictsofinterest

- Please note that all corresponding authors are required to supply an ORCID ID for their name upon submission of a revised manuscript (<https://orcid.org/>), which can be linked to your profile in the manuscript submission system. This information is currently missing for Dr. Mindong Ren.

- Reference list: please use et al after 10 author names. DOIs are only used for preprints.

- These funders are missing from the online submission system as separate entries - The Seventh District Foundation, the Laura and Isaac Perlmutter Cancer Center. Please add them.

- Please provide the figures in either of these formats: .eps, .tif, .jpg. Powerpoint figures can unfortunately not be used for production.

- Table EV1 and Table EV2 are complex datasets and need to be updated to Dataset EV1 and Dataset EV2 in all places (file names, callouts in the manuscript). Please also add the name Dataset EV# in the legend of the Dataset (separate tab of the file).

- Please upload the Reagents and Tools table as a separate .docx file. Our production team will typeset it into the article.

- If some of the sections of the Reagents and Tools table do not apply, you can simply delete them from the table, e.g., Recombinant DNA, Antibodies.

- In the Author Checklist you filled line 58, Animals observed or captured from the field, which I think does not apply to your study, as you only used transgenic mice housed in an animal facility.
- Moreover, you indicated that you have referred to external step by step protocols that are available. Can you please double-check as this would refer to published protocols at protocols.io or a methods journal.

- Ethics: please add information on the reference number for the approval of mouse work in the methods secion.

- Source data: all panels of one figure need to be combined into one figure folder so that we have separate figure folders uploaded: Figure 1 Source Data, Figure 4 Source Data.

- Source data provided for Figure 1F: please provide the photographs as individual images in a folder called "1F". Currently, the source data are a duplication of the panel.

- The paragraph SUPPLEMENTAL INFORMATION should be removed from the manuscript. It is not required.

- Materials and Methods should be Methods

- Our production/data editors have asked you to clarify several points in the figure legends (see below). Please incorporate these

changes in the manuscript and return the revised file with tracked changes with your final manuscript submission.
- Please note that the exact p values are not provided in the legends of figures 1C, G; 2B, D; 3E, 4A, B, C
- Please indicate the statistical test used for data analysis in the legends of figures 2A, B, D, 3A-E
- Please note that the box plots need to be defined in terms of minima, maxima, centre, bounds of box and whiskers, and percentile in the legend of figure 4C.
- Please note that the error bars are not defined in the legends of figure 4A.

- As a standard, we modify title and abstracts of papers to make them more accessible to our general readership. Please find my edited suggestions at the end of this e-mail.

- Finally, EMBO Reports papers are accompanied online by
A) a short (1-2 sentences) summary of the findings and their significance,
B) 2-3 bullet points highlighting key results and
C) a schematic summary figure that provides a sketch of the major findings (not a data image).
Please provide the summary figure as a separate file in PNG or JPG format at a size of 550x300 pixels (width x height). Please note that the size is rather small and that text needs to be readable at the final size. Please send us this information along with the revised manuscript.

I look forward to seeing a new revised version of your manuscript as soon as possible.

Kind regards,

Martina Rembold, PhD
Senior Editor
EMBO reports

====================

Referee #1:

The major concerns raised were properly answered.

Referee #2:

The revisions performed by Ren et al. is thorough and takes the comments from reviewers into satisfactory consideration.

A few comments for the final submission of manuscript and figures.

1. The resolution of electron microscopy pictures in figure 4 is too poor - please ensure to upload these picture in a better resolution.
2. It is fair that the authors were not able to perform additional studies, (reviewer 1, comment 6), but the authors should make this very clear in their description of the results.

====================

De novo synthesis of cardiolipin controls respiratory chain biogenesis in neonatal mouse hearts

Postnatal maturation of the mammalian heart requires a vast increase in respiratory enzymes. The mitochondria-specific lipid cardiolipin (CL) is essential for cardiac function and respiratory chain integrity but has an undefined function in heart maturation. Here, we determine how the two steps of CL biogenesis, de novo synthesis and acyl chain remodeling, affect the maturation of cardiac mitochondria in mice. Cardiomyocyte-restricted deletion of the CL synthase Crsl1 in late gestation does not affect CL levels at birth, but blocks the increase in the tissue concentration of CL observed during normal postnatal maturation. Deletion of Crsl1 prevents the postnatal rise in cristae density and in the intra-mitochondrial concentration of respiratory proteins. This inhibits cardiac development, precipitates heart failure, and causes death by the age of 2 weeks. In contrast, ablation of CL remodeling by cardiomyocyte-restricted deletion of Tafazzin, does not disrupt mitochondrial maturation or cardiac development although it has a similar effect on the CL concentration and profoundly alters the CL species composition. Our data show that CL synthesis, but not CL remodeling, controls expression of the respiratory chain by a mechanism independent of the CL concentration.

The authors have addressed all minor editorial requests.

Dr. Mindong Ren
New York University - Langone Health
United States

Dear Dr. Ren,

I am very pleased to accept your manuscript for publication in the next available issue of EMBO reports. Thank you for your contribution to our journal.

Your manuscript will be processed for publication by EMBO Press. It will be copy edited and you will receive page proofs prior to publication. Please note that you will be contacted by Springer Nature Author Services to complete licensing and payment information.

You may qualify for financial assistance for your publication charges - either via a Springer Nature fully open access agreement or an EMBO initiative. Check your eligibility: https://link.springer.com/journal/44319/how-to-publish-with-us

Should you be planning a Press Release on your article, please notify the production team via this form in order to coordinate publication and release dates: https://forms.cloud.microsoft/e/eH4A8JVP6g

If you have any questions, please do not hesitate to contact the Editorial Office. Thank you for your contribution to EMBO Reports.

Yours sincerely,

Martina Rembold, PhD
Senior Editor
EMBO reports

>>> Please note that it is EMBO Reports policy for the transcript of the editorial process (containing referee reports and your response letter) to be published as an online supplement to each paper. If you do NOT want this, you will need to inform the Editorial Office via email immediately. More information is available here: https://link.springer.com/partners/embo-press/editorial-policies#Peer%20review

EMBO Press Author Checklist

Corresponding Author Name: Mindong Ren & Michael Schlame
Journal Submitted to: EMBO Reports
Manuscript Number: EMBOR-2025-63247-T

USEFUL LINKS FOR COMPLETING THIS FORM
The EMBO Journal - Author Guidelines
EMBO Reports - Author Guidelines
Molecular Systems Biology - Author Guidelines
EMBO Molecular Medicine - Author Guidelines

Reporting Checklist for Life Science Articles (updated January
This checklist is adapted from Materials Design Analysis Reporting (MDAR) Checklist for Authors. MDAR establishes a minimum set of requirements in transparent reporting in the life sciences (see Statement of Task: 10.31222/osf.io/9sm4x). Please follow the journal's guidelines in preparing your
Please note that a copy of this checklist will be published alongside your article.

Abridged guidelines for figures
1. Data
The data shown in figures should satisfy the following conditions:
- the data were obtained and processed according to the field's best practice and are presented to reflect the results of the experiments in an accurate and unbiased manner.
- ideally, figure panels should include only measurements that are directly comparable to each other and obtained with the same assay.
- plots include clearly labeled error bars for independent experiments and sample sizes. Unless justified, error bars should not be shown for technical
- if n<5, the individual data points from each experiment should be plotted. Any statistical test employed should be justified.
- Source Data should be included to report the data underlying figures according to the guidelines set out in the authorship guidelines on Data

2. Captions
Each figure caption should contain the following information, for each panel where they are relevant:
- a specification of the experimental system investigated (eg cell line, species name).
- the assay(s) and method(s) used to carry out the reported observations and measurements.
- an explicit mention of the biological and chemical entity(ies) that are being measured.
- an explicit mention of the biological and chemical entity(ies) that are altered/varied/perturbed in a controlled manner.
- the exact sample size (n) for each experimental group/condition, given as a number, not a range;
- a description of the sample collection allowing the reader to understand whether the samples represent technical or biological replicates (including how many animals, litters, cultures, etc.).
- a statement of how many times the experiment shown was independently replicated in the laboratory.
- definitions of statistical methods and measures:
  - common tests, such as t-test (please specify whether paired vs. unpaired), simple χ2 tests, Wilcoxon and Mann-Whitney tests, can be unambiguously identified by name only, but more complex techniques should be described in the methods section;
  - are tests one-sided or two-sided?
  - are there adjustments for multiple comparisons?
  - exact statistical test results, e.g., P values = x but not P values < x;
  - definition of 'center values' as median or average;
  - definition of error bars as s.d. or s.e.m.

Please complete ALL of the questions below.
Select "Not Applicable" only when the requested information is not relevant for your study.

Materials

Newly Created Materials	Information included in the manuscript?	In which section is the information available? (Reagents and Tools Table, Materials and Methods, Figures, Data Availability Section)
New materials and reagents need to be available; do any restrictions apply?	Not Applicable	

Antibodies	Information included in the manuscript?	In which section is the information available? (Reagents and Tools Table, Materials and Methods, Figures, Data Availability Section)
For **antibodies** provide the following information:<br>- Commercial antibodies: RRID (if possible) or supplier name, catalogue number and or/clone number<br>- Non-commercial: RRID or citation	Not Applicable	

DNA and RNA sequences	Information included in the manuscript?	In which section is the information available? (Reagents and Tools Table, Materials and Methods, Figures, Data Availability Section)
**Short novel DNA or RNA including primers, probes**: provide the sequences.	Not Applicable	

Cell materials	Information included in the manuscript?	In which section is the information available? (Reagents and Tools Table, Materials and Methods, Figures, Data Availability Section)
**Cell lines:** Provide species information, strain. Provide accession number in repository **OR** supplier name, catalog number, clone number, and/**OR** RRID.	Not Applicable	
**Primary cultures:** Provide species, strain, sex of origin, genetic modification status.	Not Applicable	
Report if the cell lines were recently **authenticated** (e.g., by STR profiling) and tested for mycoplasma contamination.	Not Applicable	

Experimental animals	Information included in the manuscript?	In which section is the information available? (Reagents and Tools Table, Materials and Methods, Figures, Data Availability Section)
**Laboratory animals or Model organisms:** Provide species, strain, sex, age, genetic modification status. Provide accession number in repository **OR** supplier name, catalog number, clone number, **OR** RRID.	Yes	
**Animal observed in or captured from the field:** Provide species, sex, and age where possible.	Not Applicable	
Please detail **housing and husbandry conditions**.	Yes	

Plants and microbes	Information included in the manuscript?	In which section is the information available? (Reagents and Tools Table, Materials and Methods, Figures, Data Availability Section)
**Plants:** provide species and strain, ecotype and cultivar where relevant, unique accession number if available, and source (including location for collected wild specimens).	Not Applicable	
**Microbes:** provide species and strain, unique accession number if available, and source.	Not Applicable	

Human research participants	Information included in the manuscript?	In which section is the information available? (Reagents and Tools Table, Materials and Methods, Figures, Data Availability Section)
If collected and within the bounds of privacy constraints report on age, sex and gender or ethnicity for all study participants.	Not Applicable	

Core facilities	Information included in the manuscript?	In which section is the information available? (Reagents and Tools Table, Materials and Methods, Figures, Data Availability Section)
If your work benefited from core facilities, was their service mentioned in the acknowledgments section?	Yes	

Design

Study protocol	Information included in the manuscript?	In which section is the information available? (Reagents and Tools Table, Materials and Methods, Figures, Data Availability Section)
If study protocol has been **pre-registered, provide DOI in the manuscript**. For clinical trials, provide the trial registration number **OR** cite DOI.	Not Applicable	
Report the **clinical trial registration number** (at ClinicalTrials.gov or equivalent), where applicable.	Not Applicable	

Laboratory protocol	Information included in the manuscript?	In which section is the information available? (Reagents and Tools Table, Materials and Methods, Figures, Data Availability Section)
Provide DOI OR other citation details if **external detailed step-by-step protocols** are available.	Not Applicable	

Experimental study design and statistics	Information included in the manuscript?	In which section is the information available? (Reagents and Tools Table, Materials and Methods, Figures, Data Availability Section)
Include a statement about **sample size** estimate even if no statistical methods were used.	Yes	
Were any steps taken to minimize the effects of subjective bias when allocating animals/samples to treatment (e.g. **randomization procedure**)? If yes, have they been described?	Yes	
Include a statement about **blinding** even if no blinding was done.	Yes	
Describe **inclusion/exclusion criteria** if samples or animals were excluded from the analysis. Were the criteria pre-established? If sample or data points were omitted from analysis, report if this was due to attrition or intentional exclusion and provide justification.	Yes	
For every figure, are **statistical tests** justified as appropriate? Do the data meet the assumptions of the tests (e.g., normal distribution)? Describe any methods used to assess it. Is there an estimate of variation within each group of data? Is the variance similar between the groups that are being statistically compared?	Yes	

Sample definition and in-laboratory replication	Information included in the manuscript?	In which section is the information available? (Reagents and Tools Table, Materials and Methods, Figures, Data Availability Section)
In the figure legends: state number of times the experiment was **replicated** in laboratory.	Yes	
In the figure legends: define whether data describe **technical or biological replicates**.	Yes	

Ethics

Ethics	Information included in the manuscript?	In which section is the information available? (Reagents and Tools Table, Materials and Methods, Figures, Data Availability Section)
Studies involving **human participants**: State details of **authority granting ethics approval** (IRB or equivalent committee(s), provide reference number for approval.	Not Applicable	
Studies involving **human participants**: Include a statement confirming that **informed consent** was obtained from all subjects and that the experiments conformed to the principles set out in the WMA Declaration of Helsinki and the Department of Health and Human Services Belmont Report.	Not Applicable	
Studies involving **human participants:** For publication of **patient photos**, include a statement confirming that consent to publish was obtained.	Not Applicable	
Studies involving experimental **animals**: State details of **authority granting ethics approval** (IRB or equivalent committee(s), provide reference number for approval. Include a statement of compliance with ethical regulations.	Yes	All protocols were approved by the Institutional Animal Care and Use Committee of the NYU School of Medicine and conform to the Guide for the Care and Use of Laboratory Animals published by the National Institutes of Health (NIH). Protocol # 160406
Studies involving **specimen and field samples:** State if relevant **permits** obtained, provide details of authority approving study; if none were required, explain why.	Not Applicable	

Dual Use Research of Concern (DURC)	Information included in the manuscript?	In which section is the information available? (Reagents and Tools Table, Materials and Methods, Figures, Data Availability Section)
Could your study fall under dual use research restrictions? Please check biosecurity documents and list of **select agents and toxins** (CDC): https://www.selectagents.gov/sat/list.htm	Not Applicable	
If you used a select agent, is the security level of the lab appropriate and reported in the manuscript?	Not Applicable	
If a study is subject to dual use research of concern regulations, is the name of the **authority granting approval and reference number** for the regulatory approval provided in the manuscript?	Not Applicable	

Reporting

The MDAR framework recommends adoption of discipline-specific guidelines, established and endorsed through community initiatives. Journals have their own policy about requiring specific guidelines and recommendations to complement MDAR.

Adherence to community standards	Information included in the manuscript?	In which section is the information available? (Reagents and Tools Table, Materials and Methods, Figures, Data Availability Section)
State if relevant guidelines or checklists (e.g., **ICMJE, MIBBI, ARRIVE, PRISMA**) have been followed or provided.	Not Applicable	
For **tumor marker prognostic studies,** we recommend that you follow the **REMARK** reporting guidelines (see link list at top right). See author guidelines, under 'Reporting Guidelines'. Please confirm you have followed these guidelines.	Not Applicable	
For **phase II and III randomized controlled trials**, please refer to the **CONSORT** flow diagram (see link list at top right) and submit the CONSORT checklist (see link list at top right) with your submission. See author guidelines, under 'Reporting Guidelines'. Please confirm you have submitted this list.	Not Applicable	

Data Availability

Data availability	Information included in the manuscript?	In which section is the information available? (Reagents and Tools Table, Materials and Methods, Figures, Data Availability Section)
Have **primary datasets** been deposited according to the journal's guidelines (see 'Data Deposition' section) and the respective accession numbers provided in the Data Availability Section?	Yes	
Were **human clinical and genomic datasets** deposited in a public access-controlled repository in accordance to ethical obligations to the patients and to the applicable consent agreement?	Not Applicable	
Are **computational models** that are central and integral to a study available without restrictions in a machine-readable form? Were the relevant accession numbers or links provided?	Not Applicable	
If publicly available data were reused, provide the respective **data citations in the reference list**.	Not Applicable	
