# Supplementary material for: De novo synthesis of cardiolipin controls respiratory chain biogenesis in neonatal mouse hearts
Source: EMBO Rep. 2026 Jul 7;27(16):4653–67. doi: 10.1038/s44319-026-00864-8 (PMC13503872; doi:10.1038/s44319-026-00864-8)
Supplement: Supplementary file 2 [file 44319_2026_864_MOESM6_ESM.pdf]

Expanded View Figures

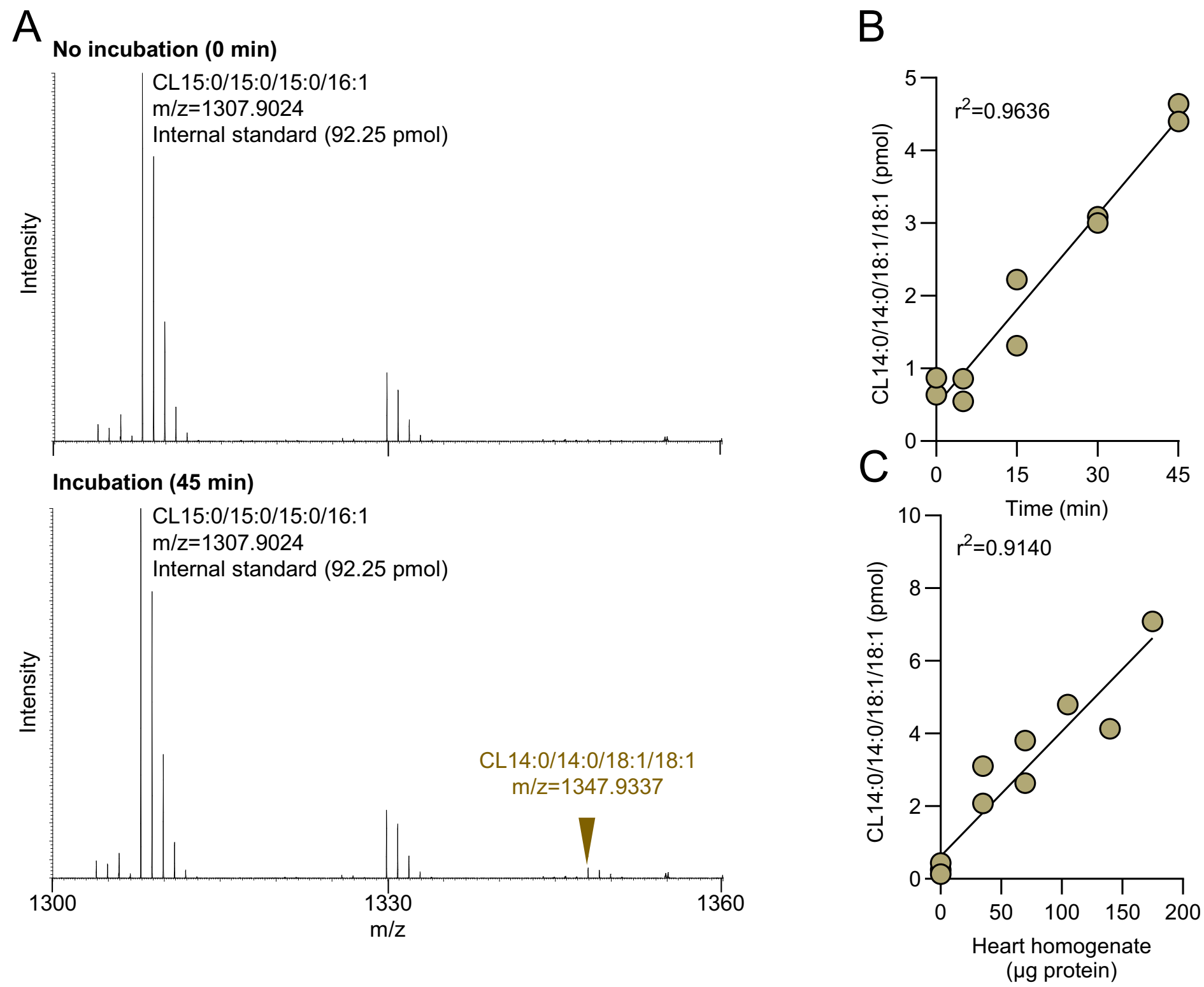

Figure EV1. Assay of cardiolipin synthase (CRLS1) activity in heart homogenate.

Mouse heart homogenate was incubated at 37 °C with dimyristoyl-phosphatidylglycerol (PG14:0/14:0) and CDP-dioleoylglycerol (CDPDG18:1/18:1) at pH 8 in the presence of Co^{2+}. The formation of CL14:0/14:0/18:1/18:1 was measured by LC–MS with internal standards. (**A**) CL14:0/14:0/18:1/18:1 is not an endogenous species of mouse heart but becomes detectable upon incubation with CRLS1 substrates. The panel shows sub-spectra (*m/z* 1300–1360 Th, retention time 12–15 min) that contain the reaction product CL14:0/14:0/18:1/18:1 and the internal standard CL 15:0/15:0/15:0/16:1. The blank signal corresponded to 0.549 ± 0.266 pmol CL14:0/14:0/18:1/18:1 ($N = 4$). (**B**) CL14:0/14:0/18:1/18:1 increases at a constant rate during incubation. Assay mixtures contained 10 mM Tris (pH 8), 5 mM $CoCl_2$, 0.1 mM PG14:0/14:0, 0.1 mM CDPDG18:1/18:1, and 70 µg heart protein in a total volume of 100 µL. Reactions were stopped after different time intervals. Lipids were extracted, and CL was quantified by LC–MS. Linear regression analysis was performed. (**C**) Formation of CL14:0/14:0/18:1/18:1 is linearly dependent on the amount of heart homogenate. Assay mixtures contained 10 mM Tris (pH 8), 5 mM $CoCl_2$, 0.1 mM PG14:0/14:0, 0.1 mM CDPDG18:1/18:1, and different amounts of heart homogenate in a total volume of 100 µL. Reactions were stopped after 30 min. Lipids were extracted, and CL was quantified by LC–MS. Linear regression analysis was performed.

© The Author(s)

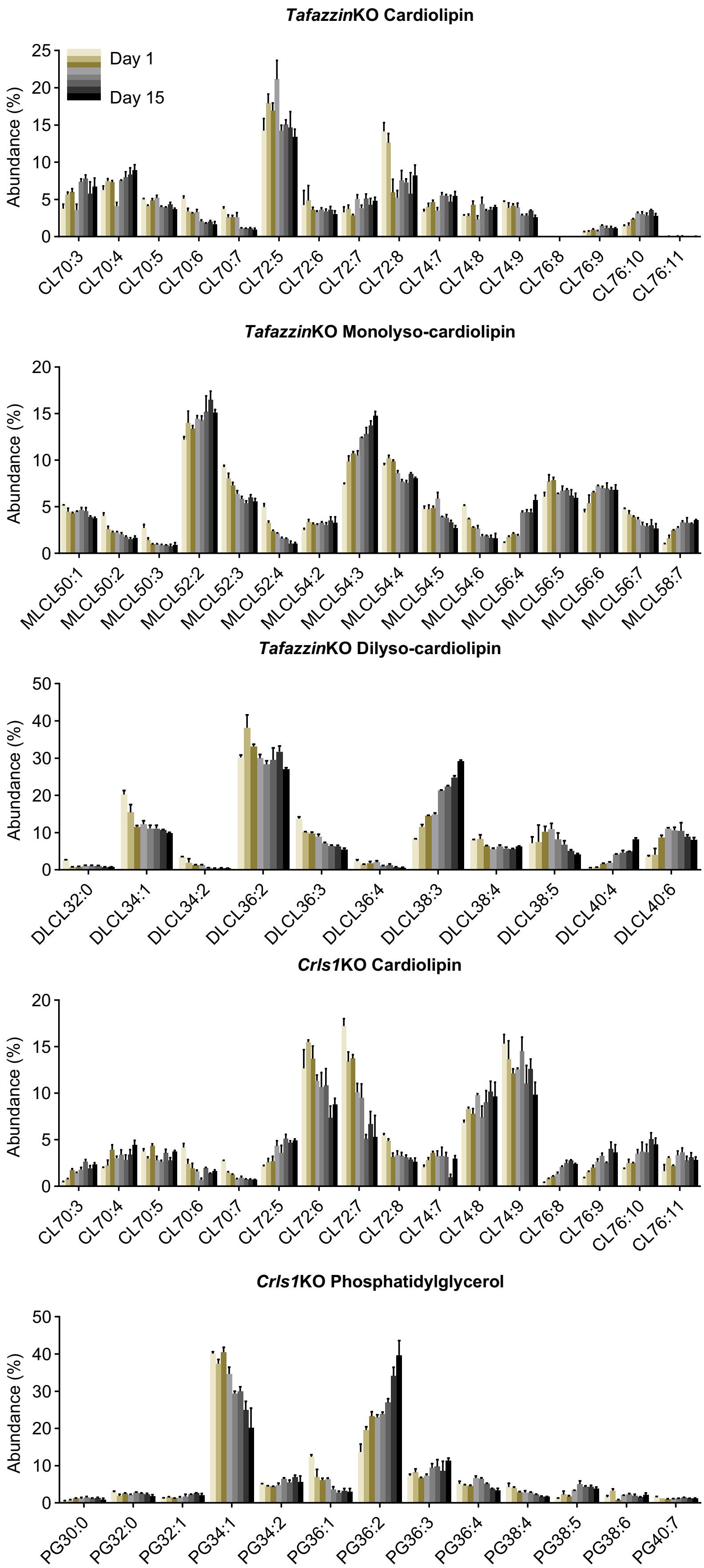
TafazzinKO Cardiolipin
Day 1
Day 15
Abundance (%)
25
20
15
10
5
0
CL70:3
CL70:4
CL70:5
CL70:6
CL70:7
CL72:5
CL72:6
CL72:7
CL72:8
CL74:7
CL74:8
CL74:9
CL76:8
CL76:9
CL76:10
CL76:11
TafazzinKO Monolyso-cardiolipin
Abundance (%)
20
15
10
5
0
MLCL50:1
MLCL50:2
MLCL50:3
MLCL52:2
MLCL52:3
MLCL52:4
MLCL54:2
MLCL54:3
MLCL54:4
MLCL54:5
MLCL54:6
MLCL56:4
MLCL56:5
MLCL56:6
MLCL56:7
MLCL58:7
TafazzinKO Dilyso-cardiolipin
Abundance (%)
50
40
30
20
10
0
DLCL32:0
DLCL34:1
DLCL34:2
DLCL36:2
DLCL36:3
DLCL36:4
DLCL38:3
DLCL38:4
DLCL38:5
DLCL40:4
DLCL40:6
Crls1KO Cardiolipin
Abundance (%)
20
15
10
5
0
CL70:3
CL70:4
CL70:5
CL70:6
CL70:7
CL72:5
CL72:6
CL72:7
CL72:8
CL74:7
CL74:8
CL74:9
CL76:8
CL76:9
CL76:10
CL76:11
Crls1KO Phosphatidylglycerol
Abundance (%)
50
40
30
20
10
0
PG30:0
PG32:0
PG32:1
PG34:1
PG34:2
PG36:1
PG36:2
PG36:3
PG36:4
PG38:4
PG38:5
PG38:6
PG40:7

© The Author(s)

Figure EV2. Age-dependent species patterns of CL, MLCL, DLCL, and PG in *Tafazzin*KO and *Crls1*KO.

Lipids were analyzed in heart homogenates at different postnatal ages. Bar graphs show means with standard deviations ($N = 3$) of abundant molecular groups (> 2% of phospholipid class).
