# Supplementary material for: De novo synthesis of cardiolipin controls respiratory chain biogenesis in neonatal mouse hearts
Source: EMBO Rep. 2026 Jul 7;27(16):4653–67. doi: 10.1038/s44319-026-00864-8 (PMC13503872; doi:10.1038/s44319-026-00864-8)
Supplement: Supplementary file 3 [file 44319_2026_864_MOESM4_ESM.zip › Figure 1 Source Data/1F/1F_TafazzinKO-P13.pdf]

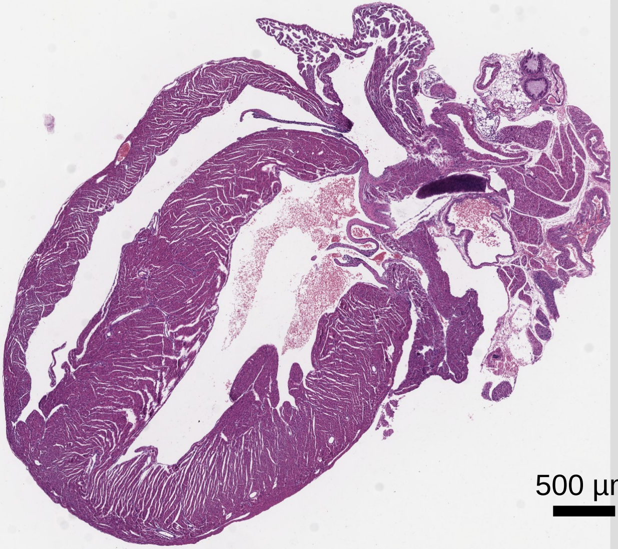
500 µm
